# Deciphering the Potential Pharmaceutical Mechanism of GUI-ZHI-FU-LING-WAN on Systemic Sclerosis based on Systems Biology Approaches

**DOI:** 10.1038/s41598-018-36314-2

**Published:** 2019-01-23

**Authors:** Qiao Wang, Guoshan Shi, Yun Zhang, Feilong Lu, Duoli Xie, Chengping Wen, Lin Huang

**Affiliations:** 1 TCM Clinical Basis Institute, Zhejiang Chinese Medicine University, 548 Binwen Road, Hangzhou, Zhejiang, 310000 China; 2 Department of Integrative Traditional & Western Medicine, Medical College, Yangzhou University, Yangzhou, Jiangsu 225001 China

## Abstract

Systemic sclerosis (SSc; scleroderma) is a complicated idiopathic connective tissue disease with seldom effective treatment. GUI-ZHI-FU-LING-WAN (GFW) is a classic Traditional Chinese Medicine (TCM) formula widely used for the treatment of SSc. However, the mechanism of how the GFW affects SSc remains unclear. In this study, the system biology approach was utilized to analyze herb compounds and related targets to get the general information of GFW. The KEGG enrichment analysis of 1645 related targets suggested that the formula is involved in the VEGF signaling pathway, the Toll-like receptor signaling pathway, etc. Quantitative and qualitative analysis of the relationship among the 3 subsets (formula targets, drug targets and disease genes) showed that the formula targets overlapped with 38.0% drug targets and 26.0% proteins encoded by disease genes. Through the analysis of SSc related microarray statistics from the GEO database, we also validated the consistent expression behavior among the 3 subsets before and after treatment. To further reveal the mechanism of prescription, we constructed a network among 3 subsets and decomposed it into 24 modules to decipher how GFW interfere in the progress of SSc. The modules indicated that the intervention may come into effect through following pathogenic processes: vasculopathy, immune dysregulation and tissue fibrosis. Vitro experiments confirmed that GFW could suppress the proliferation of fibroblasts and decrease the Th1 cytokine (TNF-α, MIP-2 and IL-6) expression for lipopolysaccharide (LPS) and bleomycin (BLM) stimulation in macrophages, which is consistent with previous conclusion that GFW is able to relieve SSc. The systems biology approach provides a new insight for deepening understanding about TCM.

## Introduction

Systemic sclerosis (SSc; scleroderma) is a complicated autoimmune disease characterized by small vessel vasculopathy, immune system abnormalities, and excessive fibrosis of the skin and internal organs. Based on the distinct difference in clinical manifestations, biological characteristics, and prognosis, SSc can be divided into 3 subsets: diffuse cutaneous SSc (dcSSc), limited cutaneous SSc (lcSSc), and SSc without skin involvement^1^. Due to its heterogeneity, the outbreak may differ in organs and manifestations, which causes the low survival in SSc. Lung involvement such as interstitial lung disease (ILD) and pulmonary arterial hypertension (PAH) lead to approximatively 50% of SSc related death^2^. Current therapeutic strategies of SSc mainly focus on the involved organs and primary symptoms, which only addresses a limited number of most relevant pharmacological issues for SSc. Moreover, low morbidity makes SSc lack of high-quality RCTs. Quite a few treatment recommendations were extrapolated from other diseases, such as ankylosing spondylitis (AS), which differs in clinical course and prognosis from SSc. In addition, toxicities/side effects are another problem need to be solved^3^. Methotrexate (MTX), an immunosuppressive agent targets the immune dysregulation, has a burden on liver function and hematopoietic system. Another example, based on evidence from several retrospective studies, patients on steroids increase SSc renal crisis (SRC) susceptibility, particularly for patients with early dcSSc^4,5^. As a result, an enhanced efficacy and minimal toxicity agent is urgently required for the treatment of SSc.

Traditional Chinese Medicine (TCM) has been used in clinical for thousands of years, experiencing repeated clinical practice and refinement, and resulted in a lot of effective prescriptions. Especially in the treatments of complex diseases, multi-component, multi-target, multi-pathway comprehensive regulations of the human body therapy is superior to the single target therapies. The prospect of TCM treatment has received more and more attention. For example, Compound Danshen dripping pills (CDDPs) have been prescribed for more than 450 million angina pectoris patients cumulatively since it came into the market in 1994. The Phase II clinical trial of Food and Drug Administration (FDA) suggested that CDDP was an effective and safe agent for the angina pectoris patients^6^.

GUI-ZHI-FU-LING-WAN (GFW) is a classic Chinese formula developed by Zhang Zhongjing in Han Dynasty who is honored as sage in history of TCM. It has been administered to the patients with SSc and many other clinical conditions, such as endometriosis, dysmenorrhea and so on^7^. It can improve the skin manifestations in the treatment of scleroderma and reduce the severity of Raynaud’s phenomenon^8^. Previous studies indicate GFW can improve skin sclerosis and rise the skin temperature^9,10^. The corresponding Chinese patent drug—GFW capsules are currently being assessed for the efficacy, safety, and dose-response on the treatment of primary dysmenorrhea by the FDA (ClinicalTrials.gov ID of FDA: NCT01588236). GFW consists of 5 herbs: Cinnamomum cassia (GUI ZHI), Poria cocos (FU LING), Semen persicae (TAO REN), Paeonia albiflora (CHI SHAO), Cortex moutan (MU DAN PI), working together to promote blood circulation and remove blood stagnation. Pharmacological research indicated that the main effect of the prescription is to dilate blood vessels, anticoagulation, anti-fibrosis and anti-inflammatory^11^. For example, Cinnamomum cassia dilates vascular smooth muscle^12,13^, Semen persicae intervene the process of hemorheology, plasma coagulation and platelet aggregation^14^, Paeonia albiflora and Cortex moutan possess the anti-fibrotic and immuno-regulatory activities^15,16^. The function of the singular herb has been explored extensively. However, such traditional research methods are usually the simple superpositions of formula compounds which is not helpful in understanding the interaction between each component and target on the whole prescription and go against the holistic concept of TCM.

The systems biology approach, which integrates multiple systems to study the biological progress on a global level, is consistent with the essence of TCM—Holism, thus it gives a new perspective to make the clarification of the GFW’s molecular mechanism. For example, interactome, as one of its fundamental discipline, is defined to investigate the interactions among various molecules to predict protein function, disease genes, and multiple aspects of drug development as an integrated system^17,18^. Correspondingly, a large number of databases and efficient network algorithms appear and greatly contribute to the development of systems biology. Free academic resources, such as TCMID (http://www.megabionet.org/tcmid/)^19^, BioGRID (https://thebiogrid.org/)^20^, OMIM (http://www.omim.org/)^21^ and so on, enable the systems biology approach to get access to the protein, genetic and chemical interactions data for the medical study. In our previous researches, we have applied systems biology approach to explore the molecular mechanism of series classic traditional formulas. For instance, the pharmaceutical mechanism of Gui-Zhi-Shao-Yao-Zhi-Mu (GSZ) decoction on rheumatoid arthritis mainly relates to multiple essential biological and immune pathways such as inflammatory reaction, treatment response of rheumatoid arthritis, etc.^22^. The targets of Sheng-ma-bie-jia-tang (SMBJT) can be defined as essential targets and common targets. The essential targets have the direct relation with the Systemic Lupus Erythematosus (SLE) disease genes, while the common targets play distinct functions in immune system processes of SLE (e.g. toll-like receptor signaling pathway)^23^.

In this paper, we aimed to decipher the mechanism of GFW formula on SSc by the integration analysis. To exhibit the whole picture of GFW on scleroderma treatment, we systematically investigate all available formula compounds and related targets. Comprehensively compare the interactions among 3 subsets (GFW targets, SSc drug targets and SSc disease genes), we further to confirm the quantitative relationship with microarray data. SSc related PPIs network construction and functional module analysis unearth the interactions between genes. The systems biology approach was utilized to explore the molecular mechanism underlying the prescription, and this method will identify a new area for the investigation of TCM.

## Results

### The herbs, compounds and targets of the GFW formula

**Figure 1:**
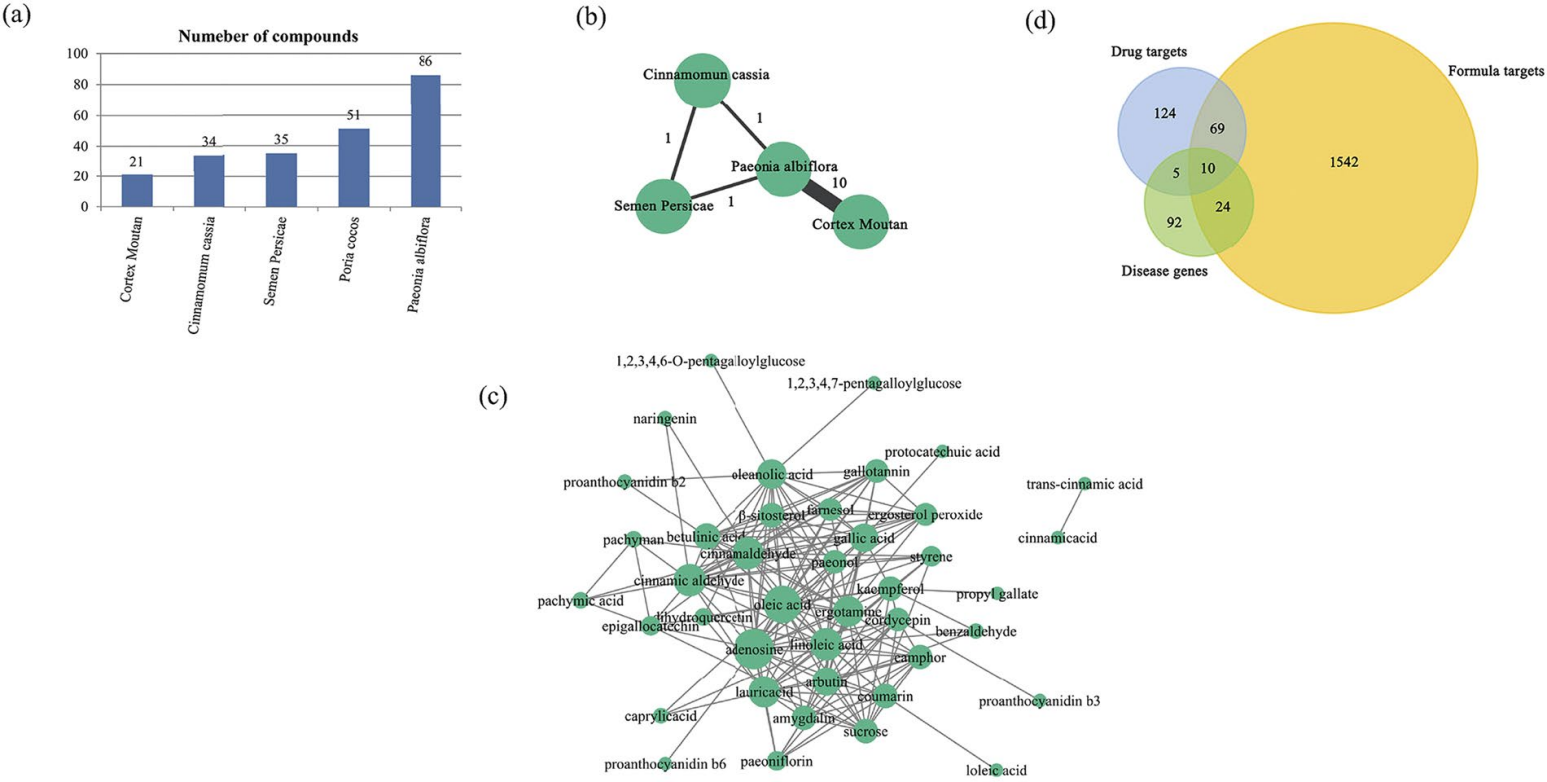
The descriptions of herbs, compounds and targets. (**a**) The detailed number of compounds in each herbs of GFW formula. (**b**) The 4 herb association pairs shared with the same compounds, the width of the edge represents the number of the common compounds. (**c**) The network is constructed with compounds sharing the same targets between compounds. The size of the node represents the number of the shared common targets. (**d**) Distribution of targets in GFW formula, drug targets and disease genes.

GFW consists of 5 kinds of herbs, namely Cinnamomum cassia, Poria cocos, Semen persicae, Paeonia albiflora, Cortex moutan. These 5 herbs connect with each other closely, exert the effect of invigorating blood, dissolving stasis, and resolving masses. A general understanding of the formula compositions is indispensable for the following analysis, so we collected all compounds related to the 5 herbs from TCMID database and finally got 214 different kinds of herbal compounds (Supplement Table S1, Fig. 1a). Among them, the representative compounds show their functions of herbs. In details, the main substance essential oil in Cinnamomum cassia responds to the pharmacological effect, which has the properties of antinociceptive and anti-inflammatory actions^24^; the poria polysaccharides and their derivatives have anticancer, antioxidant, immune-regulatory and antiviral properties^25^; Semen persicae contains a lot of fatty oil, such as palmitic acid and oleic acid, which can inhibit platelet aggregation, anticoagulant activities^26^; paeoniflorin is the most abundant compound of total paeony glucoside from Paeonia albiflora, which is identified to have antithrombotic effect^27^, anti-inflammatory and immunomodulatory effects^28^ and the phenolic compound in Cortex moutan—paeonol possess the beneficial biological activities of antioxidant, anti-inflammatory, and anti-fibrotic^29^.

Among these 214 compounds, 201 of them belong to individual herb, and the remaining 13 compounds come from more than one herbs, suggesting the synergetic functions in the biological progress. Except for Poria cocos, the other 4 herbs are composed of 4 herb association pairs according to the common constituents between the herbs (Fig. 1b). In these 4 herbs, Cortex moutan and Paeonia albiflora share 10 compounds, including paeonol, paeonoside, paeoniflorin, etc, which are related to ameliorate autoimmune and inflammatory diseases^15,30^. The common component of Cinnamomum cassia and Semen persicae—β-sitosterol has the anti-fibrotic property and regulate the release of prostacyclin, which is recommended to the management of SSc-related RP, digital ulcers and PAH^5,31–33^.

Next, we got 1645 targets related to 214 compounds from TCMID. Among these 1645 targets, 1131 targets belong to a unique compound, and the remaining 514 are shared by at least 2 compounds. The two types of targets play different roles in the formula respectively. The shared targets play synergistic roles. For instance, the targets Interleukin-6 (IL-6) and matrix metalloproteinases1 (MMP1) shared by 4 compounds can be regarded as the therapeutic targets in systemic sclerosis (SSc), the former induce chronic inflammation, endothelial abnormality and fibroblast activation which are crucial to SSc while the latter involves in the process of tissue remodeling, increasing the risk of idiopathic pulmonary fibrosis^34–36^. The 1131 targets belong to unique compound play distinct roles. For example, the unique target connective tissue growth factor (CTGF) and fibronectin 1 (FN1) are the principal mediators involving in the fibrotic process of scleroderma^37^. Angiotensin converting enzyme (ACE) is the only target of acetic acid and the inhibitors can improve survival and discontinue dialysis of scleroderma renal crisis (SRC)^3,38^. The functions of shared targets and unique targets in GFW are complementary and work together.

To further explore the compounds that play a major role in the synergy mechanism of GFW, a compound association network (Supplement Table S2, Fig. 1c) was built based on the shared targets. This compound association network was concentrated by the close connection between 5 compounds: amygdalin; arbutin; camphor; coumarin; adenosine (Supplement Fig. S1). Coumarin has antiplatelet and antithrombotic effects, which can be used as the therapeutic intervention for the SSc patients with the predisposition of thrombophilia^39–41^. In addition, the compounds separated from the center cluster also exert the effect in the formula. For example, kaempferol can inhibit fibro proliferative abnormalities via blocking TGF-β1/Smads signaling pathway^42,43^. The compound ergosterol is the source of vitamin D, which can reduce skin fibrosis in scleroderma^44,45^. In a word, we’ve acquired the information of GFW formula, compounds and targets. Next we will systematically analyze their specific function in the treatment of SSc

### KEGG pathway enrichment analysis of GFW targets

To better make sense of the functional contributions of the formula targets to the treatment of SSc, pathway enrichment analysis was carried out for the 1645 targets. Pathway enrichment analysis showed these targets were enriched in 136 pathways with P-value ≤ 0.05 (Supplementary Table S3). It is worth noting that the enrichment of the targets cover 3 crucial aspects of pathophysiological changes in SSc. VEGF signaling pathway (p = 1.00E-06), HIF-1 signaling pathway (p = 1.30E-04), Vascular smooth muscle contraction (p = 1.10E-07), Platelet activation (p = 2.90E-02) are involved in the process of scleroderma microvasculature^46^. Arginine and proline metabolism (p = 1.90E-04), PPAR signaling pathway (p = 2.10E-12), Toll-like receptor signaling pathway (p = 2.80E-03) correlate with the pro-fibrotic process of scleroderma^47,48^. Biosynthesis of antibiotics (p = 1.10E-04), T cell receptor signaling pathway (p = 2.40E-02), NOD-like receptor signaling pathway (p = 5.40E-03) drive the immune disorder progression^49^. In details, vascular endothelial growth factor (VEGF) is a proangiogenic marker and the oxygen-regulated α-subunit of hypoxia-inducible transcription factor-1 (HIF-1α) can upregulate the transcription of VEGF throughout the disease process. The concentration of VEGF can be regarded as the compensatory for endothelium disorder although it has little effect on efficient neovascularization in SSc. Moreover, serum VEGF levels can reflect interstitial lung involvement in SSc^50,51^; Toll-like receptor signaling pathway not only intervene the process of innate immunity system which contributes to both the onset and progression of SSc, but also involves in the pathogenesis of fibrosis through augmenting transforming growth factor-β1 (TGF-β1) response to increase matrix production and progressive connective tissue remodeling^52,53^. Pathway enrichment analysis results indicate that GFW shows efficacy in the treatment of scleroderma by covering 3 aspects of the pathological process: vascular abnormality, immune dysregulation and tissue fibrosis. On the whole, the TCM formula—GFW could be regarded as a synergistic therapeutic option which takes pharmacological actions by affecting multiple signaling pathways and different molecules rather than single one.

### Comparison between GFW targets, other SSc drug targets and SSc disease related proteins

To further predict the pharmaceutical mechanisms of GFW formula, we compared its 1645 targets with the 208 targets of 42 FDA approved SSc drugs (Supplement Table S4) and 131 reported SSc disease proteins (Supplement Table S5) (Fig. 1d). We noticed that 79 and 34 formula targets overlap with SSc drug targets and disease genes respectively, while 10 formula targets were both SSc disease genes and drug targets (Supplement Table S6).

**Figure 2:**
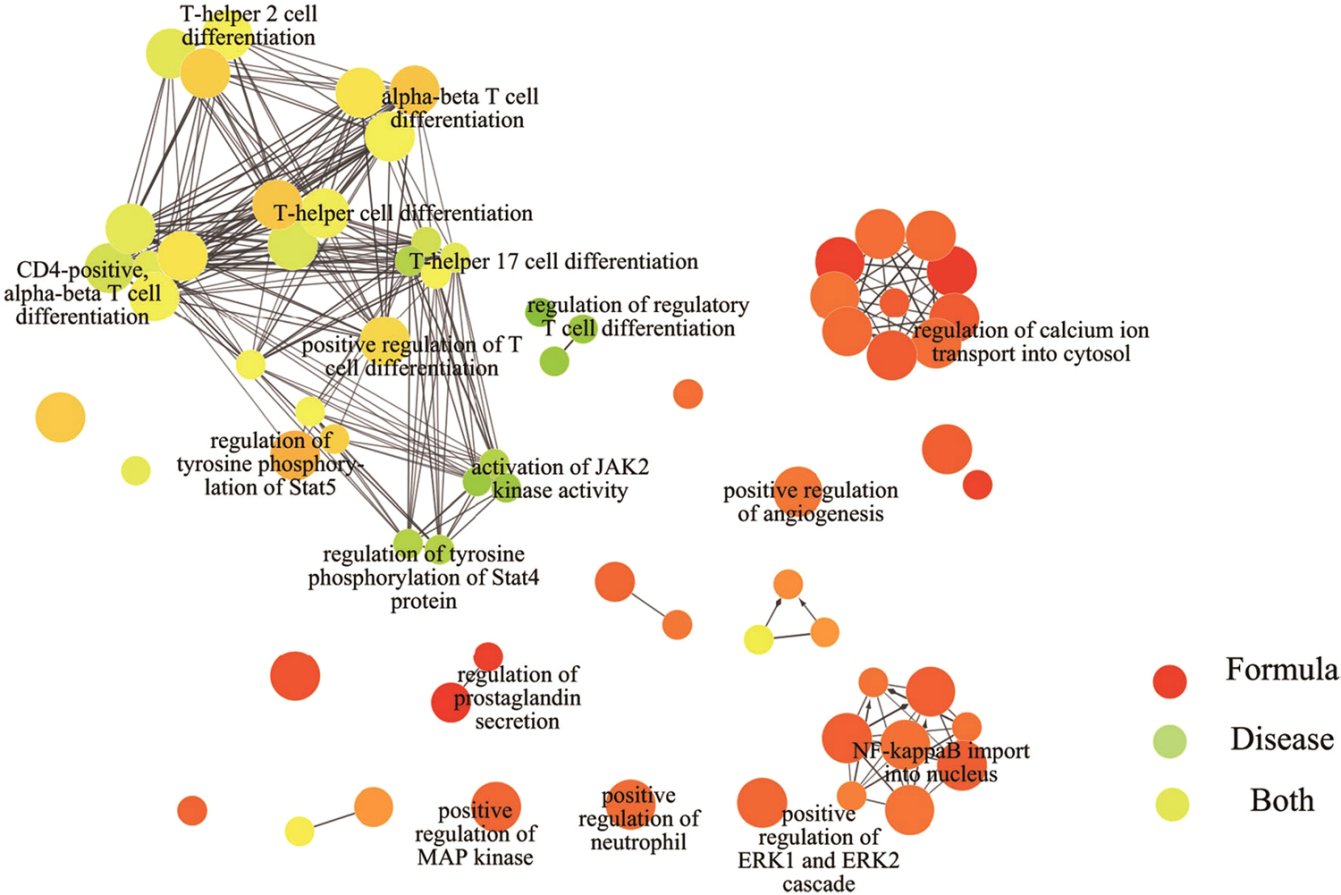
Relationship between formula targets and disease genes. The ratio of formula targets and disease genes in biological processes. The network was generated with ClueGO in Cytoscape.

34 formula targets overlapped with proteins encoded by SSc disease genes. To observe the functional connectivity between GFW formula targets and SSc disease genes, we applied ClueGO in Cytoscape to investigate the biological processes of involved genes (Fig. 2). The formula targets mainly participate in the SSc pathological process of inflammation and vascular dysfunction: positive regulation of ERK1 and ERK2 cascade, positive regulation of neutrophil chemotaxis, NF-kappaB import into nucleus, positive regulation of MAP kinase activity, positive regulation of angiogenesis, regulation of prostaglandin secretion while the disease genes mainly involve in the immunity alterations: regulation of regulatory T cell differentiation, regulation of tyrosine phosphorylation of Stat4 protein, activation of JAK2 kinase activity. The shared targets cover the acquired immunity aspects: T-helper 17 cell differentiation, regulation of T-helper 2 cell differentiation, T-helper cell differentiation, T-helper 2 cell differentiation, which indicates that the formula targets might take effect through regulation of associated immunocytes. Studies show that T helper cell 2 (Th2), T helper cell 17 (Th17), regulatory T (Treg) cell and their associated cytokines play pivotal roles in the pathogenesis of SSc^54^. GFW may regulate Th17/Tregs cells balance, Th1/Th2 cells responses and secretion of related cytokines such as shared targets IL-10, IL-6, IL-13 to reduce the autoimmune inflammatory reaction^55^. For example, the shared targets IL-6, MCP-1 belong to GFW targets and disease genes are produced by interleukin 17 (IL-17) and Th17 cells, which are increased in the peripheral blood and target organs of SSc. We predict GFW may inhibit the interleukin 17 (IL-17) and Th17 cells to relieve SSc by reducing the 2 above overlapping targets^56^.

**Figure 3:**
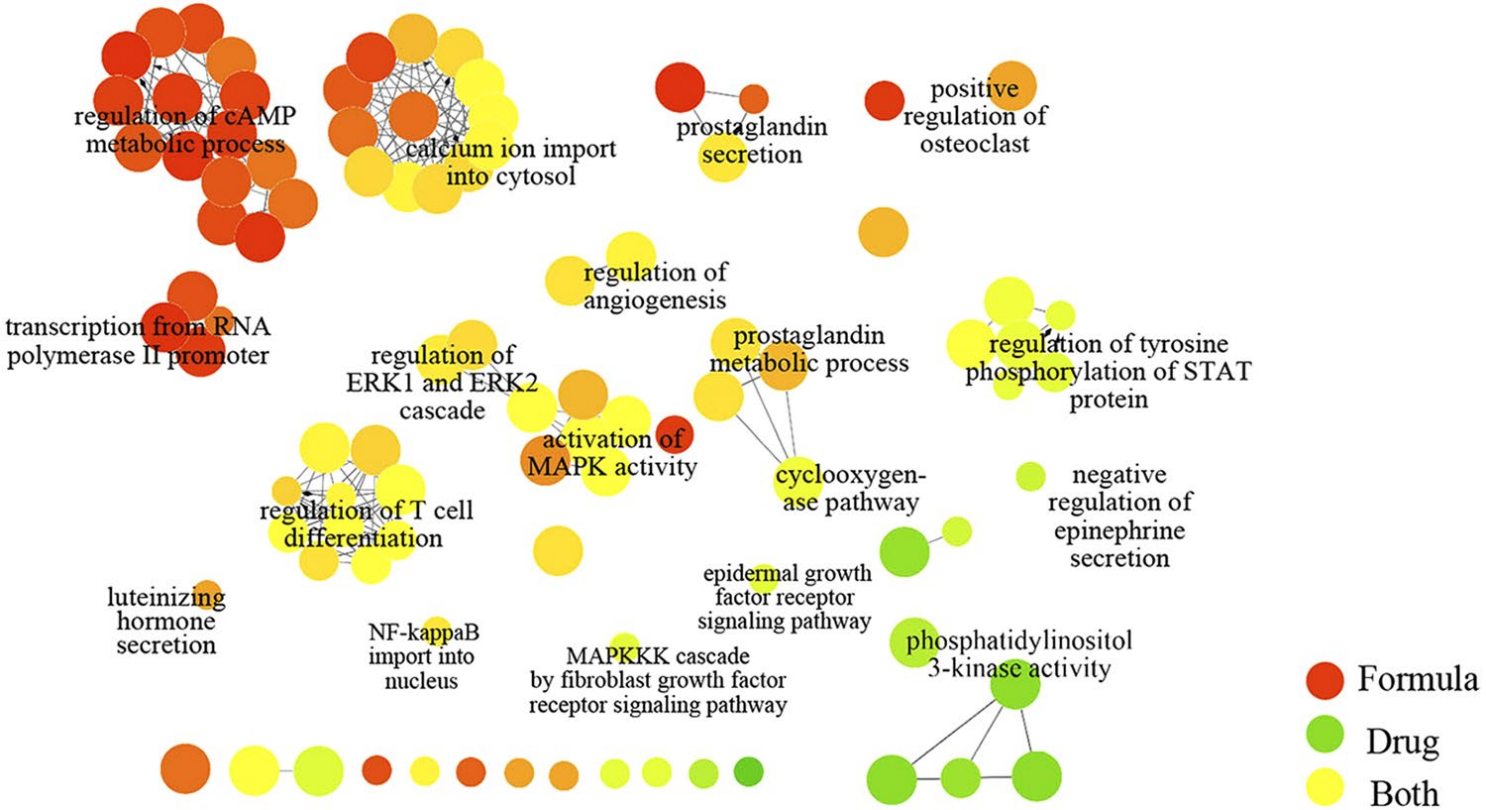
Relationship between formula targets and drug targets. The ratio of formula targets and drug targets in biological processes. The network was generated with ClueGO in Cytoscape.

Almost 38.0% FDA approved SSc drug targets overlapped with formula targets. The shared targets Endothelin B receptor (EDNRB) and Endothelin-1 receptor (EDNRA) belong to Bosentan, which can ameliorate digital ulcers and PAH on SSc patients. Inosine-5′-monophosphate dehydrogenase 1 (IMPDH1) and Inosine-5′-monophosphate dehydrogenase 2 (IMPDH2) are attributed to mycophenolate mofetil, which can treat skin involvement^3^. As illustrated in Fig. 3, the shared targets could directly affect the functional drug targets by participating in the regulation of T cell differentiation, regulation of MAPK activity, regulation of angiogenesis, and regulation of ERK1 and ERK2 cascade. Moreover, the formula targets could take effects indirectly by connecting with the drug targets, e.g. with the formula targets, cyclooxygenase pathway is extended to prostaglandin metabolic process. The formula and drug targets seem to be involved in the distinct progress. Formula targets participate in the regulation of cAMP metabolic process, calcium ion import into cytosol, prostaglandin secretion, while drug targets are related to the tyrosine phosphorylation of STAT protein, phosphatidylinositol 3-kinase activity, epidermal growth factor receptor signaling pathway, negative regulation of epinephrine secretion. The combination of two parts makes the treatment more systematic and comprehensive.

### PPIs networks predicting the mechanism of GFW treatment

**Figure 4:**
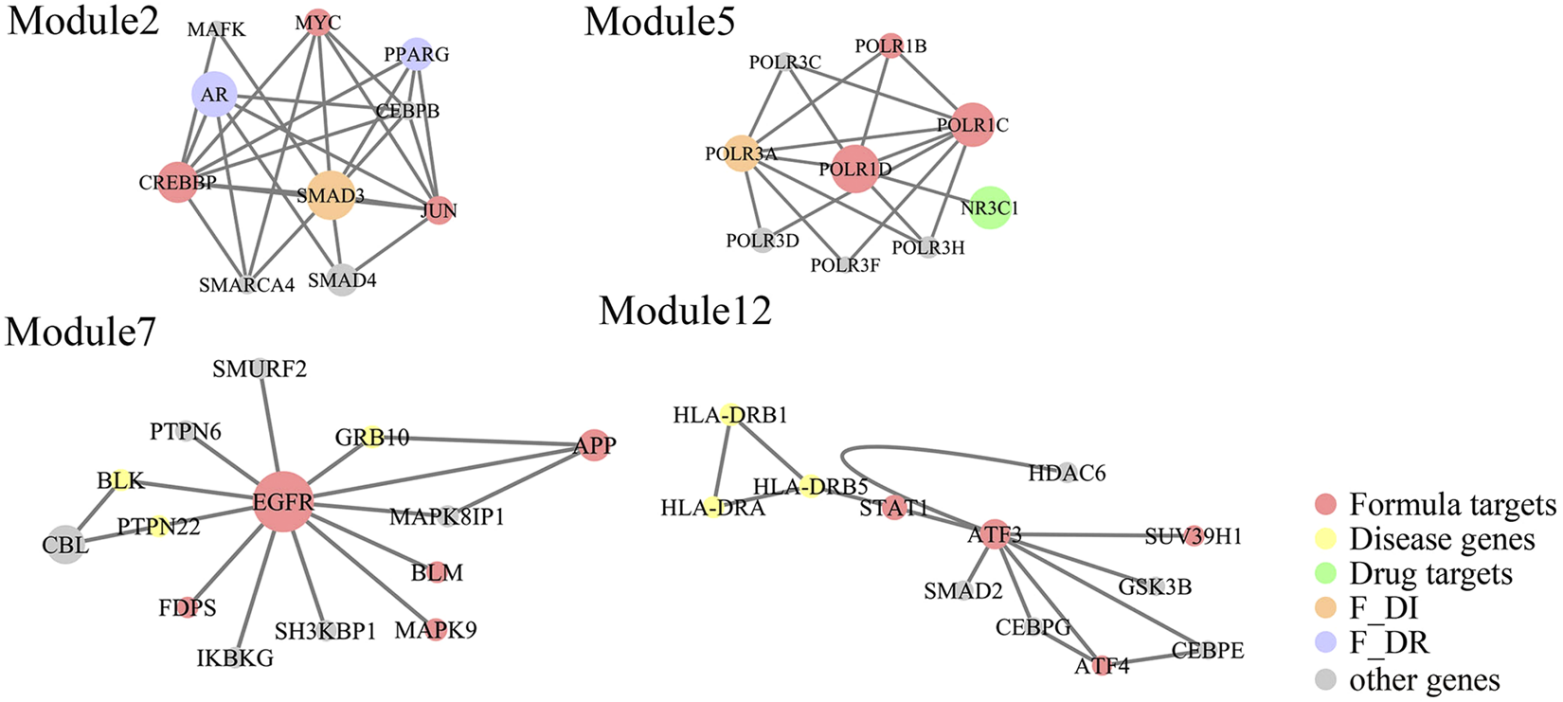
The PPI network with GFW formula targets, disease genes, drug targets and other targets. The network was constructed by ClueGO in Cytoscape. (red: Formula targets; yellow: Disease genes; green: drug targets; orange: Formula targets and disease genes; purple: Formula targets and drug targets; gray: other genes).

Data integration and visualization facilitate the dig of meaningful information from the large volume of heterogeneous network. Accordingly, to further decipher the molecular mechanism of GFW formula, we integrated formula targets, disease genes, drug targets and focused on the network analysis. A protein-protein interaction (PPI) network consisting of 12443 nodes and 104604 edges was constructed based on the formula targets, drug targets and disease genes. Then, we decomposed the network into 24 functional modules (Fig. 4, Supplement Fig. S2). The modules presented as molecular complexes which generally gathered together according to the same biological function. Each module was carried out to get functional annotation—Gene Ontology (GO) terms by enrichment analysis. Finally, we obtained 126 GO terms according to P-value ≤ 0.05 (Supplement Table S7)^57^. Module 1, Module 2, Module 6, and Module 12 involved in the fibrotic process, including TGF-β receptor signaling pathway, positive regulation of epithelial to mesenchymal transition, etc; Module 4, Module 5, Module 8, Module 9, and Module 10 were bound up with immune disorder, includes positive regulation of B cell proliferation, innate immune response, etc; Module 3, Module 7, and Module 11 are related to the microangiopathy process, such as platelet activation, vascular endothelial growth factor receptor signaling pathway and so on. The rest modules mainly act on non-specific biochemical function such as cell differentiation, protein serine/threonine phosphatase activity, etc.

The functional modules analysis of PPI network exhibited the pathological relevance of the 3 subsets, implying GFW targets take pharmacological effects through interacting with SSc related disease genes and drug targets directly or indirectly. Many lines of evidence demonstrate distinct roles of formula targets under SSc pathogenic conditions, which work by interfering with 3 parts of representative processes: immune disorder, vasculopathy and tissue fibrosis. In the first part, GFW takes effect in the fibrotic process of SSc through interacting with disease genes, drug targets. For example, Smad3 defined as the core molecule in module 2 interacts with 9 different genes. It is evident that it can be regarded as a pivotal intracellular signal transducer involving in classic pro-fibrotic TGF-β/Smad3 signaling pathway^58^. Among the 9 connected genes, peroxisome proliferator-activated receptor gamma (PPARG, drug target of pioglitazone and rosiglitazone) directly interacts with the hub node, defined as both GFW target and drug target. Its expression defect carries a higher risk of SSc, which attributes to suppress the collagen synthesis abnormalities, myofibroblast differentiation alterations, and other TGF-β-induced fibrotic responses^59^. Another drug related target Smad3 indirectly interacts with is AR (drug target of spironolactone). In addition, formula target JUN promotes the activation of SSc fibroblasts, while Smad4 is a signaling partner facilitates transducing TGF-β signals^60^. In module 12, activating transcription factor 3 (ATF3) and signal transducer and activator of transcription 1 (STAT1) are the core nodes. The formula target ATF3 not only regulates oxidation and cellular stress response, but also participates in the TGF-β signaling in SSc^61^. The other indispensable formula target STAT1 has been demonstrated to be blocked in the fibroblasts from scleroderma-associated interstitial lung disease^62^. Moreover, it takes part under certain conditions by connecting with 3 SSc-related MHC genes: HLA-DRB1, HLA-DRB5, HLA-DRA, which associate with the auto-antibody profiles, general susceptibility to SSc, disease subtypes, or certain SSc clinical features^63,64^. Taken together, the evidence indicates formula targets play a synergistic role of blocking the fibrosis progression in SSc by interacting with the disease genes and drug targets directly or indirectly.

Secondly, PPI network indicates GFW formula function as a therapeutic role in microangiopathy process. For example, in module 7, epidermal growth factor receptor (EGFR), a formula target, has been confirmed to correlate with vascular smooth muscle cells remodeling in SSc^65^. It connects with 3 SSc-associated polymorphic genes BLK, PTPN22 and GRB10. The BLK (B lymphocyte kinase) gene codes for B lymphocyte kinase, which may disrupt gene expression in B cells especially via the nuclear factor kappa B (NF-κB) signaling pathway^66^. The PTPN22 gene encodes the protein tyrosine phosphatase lymphoid tyrosine phosphatase in T cells and performs to block T cell signaling through dephosphorylation of substrates^2^. GRB10 gene encodes growth factor receptor-bound protein 10 is defined as an adaptor protein interacting with several tyrosine kinase receptors^67^. There are many other genes indirectly connect with the hub genes EGFR to expand its therapeutic effect in SSc. For example, the high degree genes CBL (Cbl Proto-Oncogene) has not yet been confirmed in the pathophysiology of SSc, however, it relates to the matrix synthesis and cellular interaction^68^. Above all, in this part, the formula target EGFR extends the function of the single pathological field through the connected disease genes and drug targets. It takes effect in the field of vascular abnormality in parallel to the immune disorder and fibrosis. Thus, these findings imply TCM formula exerts an optimal therapeutic effect by targeting multi-targets and multi-pathways.

Thirdly, we focus the function of formula on the SSc related pathological process: immune disorders. The node POLR1D in module 5 works through connecting with the other 8 nodes. The shared drug target NR3C1 (drug targets of prednisone, spironolactone and Prednisolone) encodes the receptor for glucocorticoids (GCs), which exerts anti-inflammatory and immunosuppressive functions by binding to GC receptors^69^. Another important node POLR3A, defined as both the GFW target and disease gene, also involves in SSc acquired immunity condition. The mutation in POLR3A could cause tissue injury in SSc patients by triggering cellular and humoral immune responses^70^. The rest of nodes are also factors contributing to the immune process, for example, POLR3H, POLR3F belong to the POLR3 complex, have been demonstrated the mutations could induce autoimmunity recently^71^.

In addition, the formula targets separated from the modules also show efficiency in the formula. For example, CCR6 polymorphisms associate with the higher risk of SSc^72^.

### Validation of GFW targets in large scale expression profiles of SSc patients

**Figure 5:**
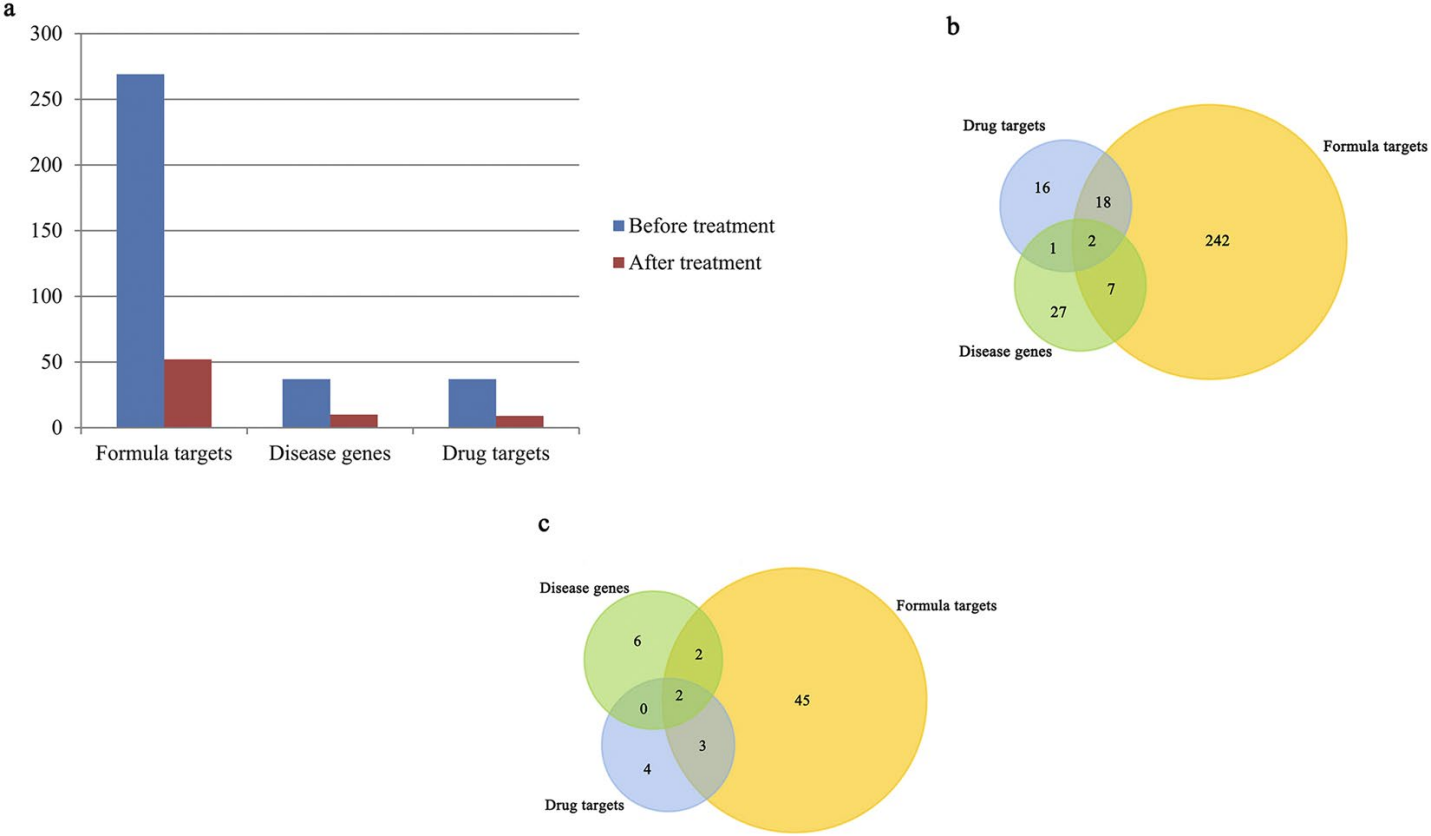
Quantitative comparison of GFW targets, drug targets and SSc disease genes. (**a**) The numbers of DEGs before and after treatment. (**b**) The distribution of GFW targets, drug targets and SSc genes before treatment. (**c**) The distribution of GFW targets, drug targets and SSc genes after treatment.

In addition to interaction networks, quantitative analysis can also reflect the relationship among the formula targets, drug targets and disease genes. We use the gene expression profiles of SSc from 121 specimens (15 normal, 61 SSc disease, 45 SSc treatment) downloaded from GEO database. The Limma package was utilized to pick out the differentially expressed genes (DEGs), all of which decreased after treatment in the 3 datasets (Fig. 5a). Specifically, DEGs in formula targets before and after drug treatment are respectively 269 and 52, dropping off 80.7%, while the drug targets and disease genes are 75.7% and 73.0%, which exhibits a similar downward trend. The consistent expression behavior may indicate that the 3 data sets may possess the similar functions. In addition, we found that the proportion of the changed drug targets and disease genes in GFW formula targets increased. Before the treatment, 24.3% (9/37) disease genes and 54.0% (20/37) drug targets belong to formula targets, while after treatment, the proportion turned to 40% (4/10) and 55.5% (5/9). It may indicate GFW formula covers the most effective biological function of other drugs and SSc related disease genes.

### GFW treated SSc by targeting fibroblast proliferation and suppressing cytokine expression in macrophages

Vitro experiments were then designed to verify the prediction of pharmacological mechanism. As mentioned, the GFW takes effect in SSc by multi-component, multi-target, multi-pathway comprehensive regulation. There is strong evidence that immune imbalance and fibroblast dysfunction contribute to the progression and perhaps onset of SSc^53,73,74^. Therefore, in this study, we focused on verifying the role of GFW in immune dysregulation and tissue fibrosis of scleroderma.

**Figure 6:**
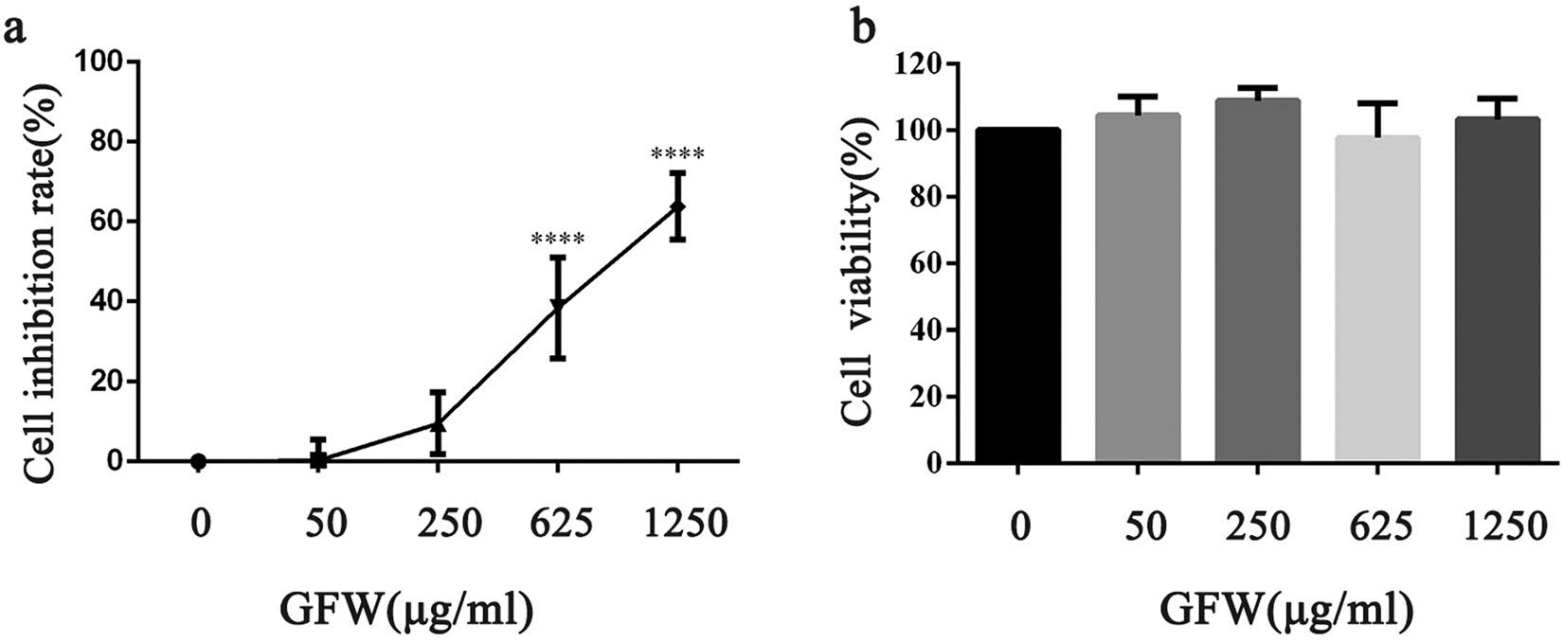
Effect of GFW on the viability and cytotoxicity of MRC-5 cells. MRC-5 cells incubated with different concentrations (0, 50, 250, 625, and 1250 μg/ml) of GFW freeze-dried powder for 48 h. (**a**) The cell inhibition rate of MRC-5 cells was detected using CCK8 assay. (**b**) The cell viability of MRC-5 cells was measured by LDH release assays. ****P < 0.0001.

Firstly, to confirm the effect of GFW in fibrosis process of SSc, the CCK8 assay was conducted *in vitro*. MRC-5 human lung fibroblast were treated by different concentrations (0, 50, 250, 625, and 1250 μg/ml) of GFW freeze-dried powder for 48 hours. The cell inhibition rate of MRC-5 treated with medium was taken as 0%. At concentration of 50, 250, 625, 1250 μg/ml, the GFW inhibited the proliferation of the MRC-5 cells by 0.32%, 9.52%, 38.27%, and 63.79% respectively (Fig. 6a). The functions of GFW inhibit the proliferation of fibroblasts should be based on the safety of the formula. The cytotoxicity of GFW was measured by LDH release assays. As shown in (Fig. 6b), there was no significant changes in cells treated with varying concentrations (0, 50, 250, 625, and 1250 μg/ml) after 48 h of incubation of GFW freeze-dried powder. Therefore, the GFW treatment can inhibit the proliferation of MRC-5 cells without cytotoxicity at all tested concentrations.

**Figure 7:**
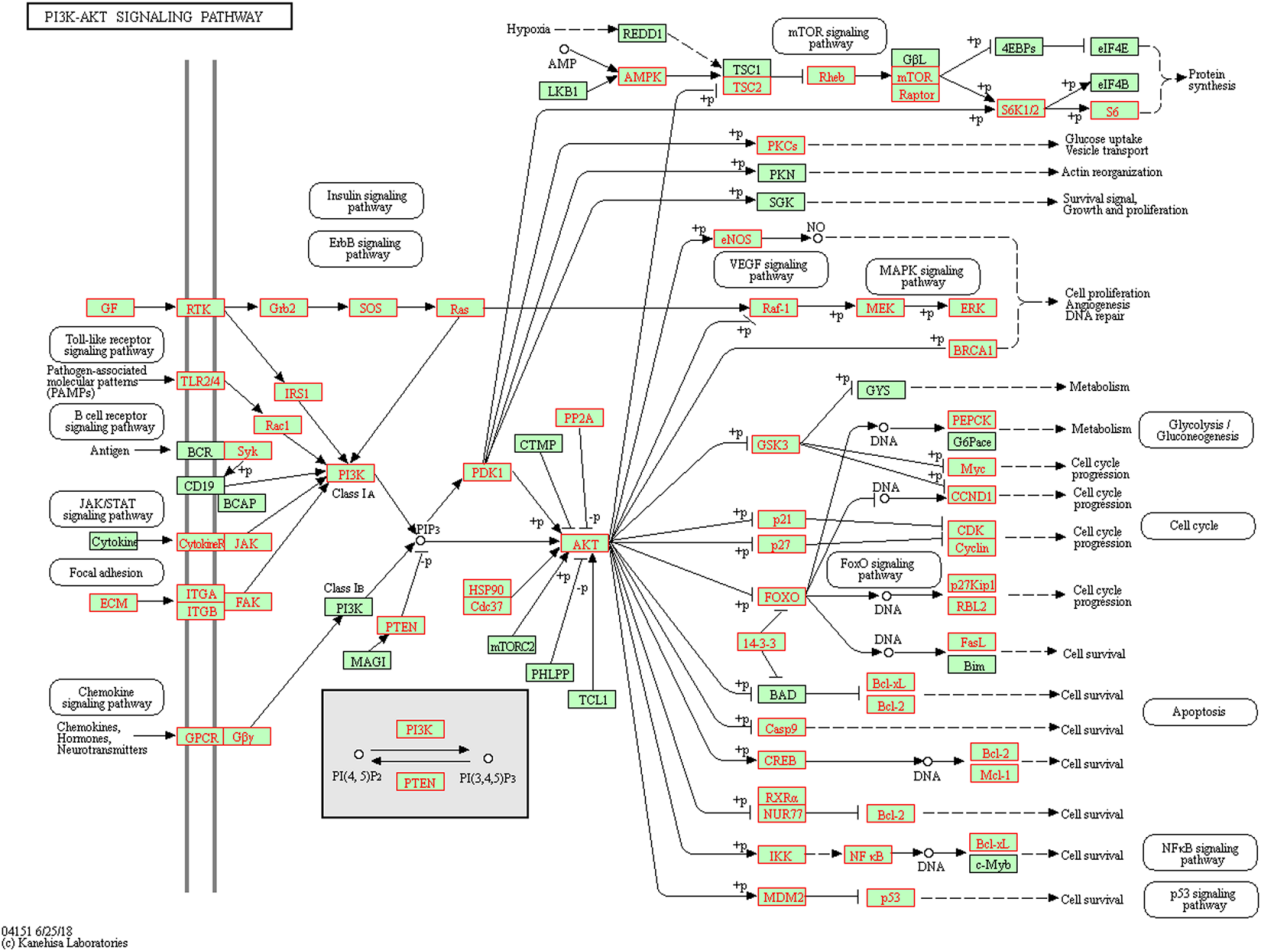
The KEGG enrichment analysis of PPIs networks downloaded from online website KEGG (https://www.kegg.jp/kegg/)^89^.

**Figure 8:**
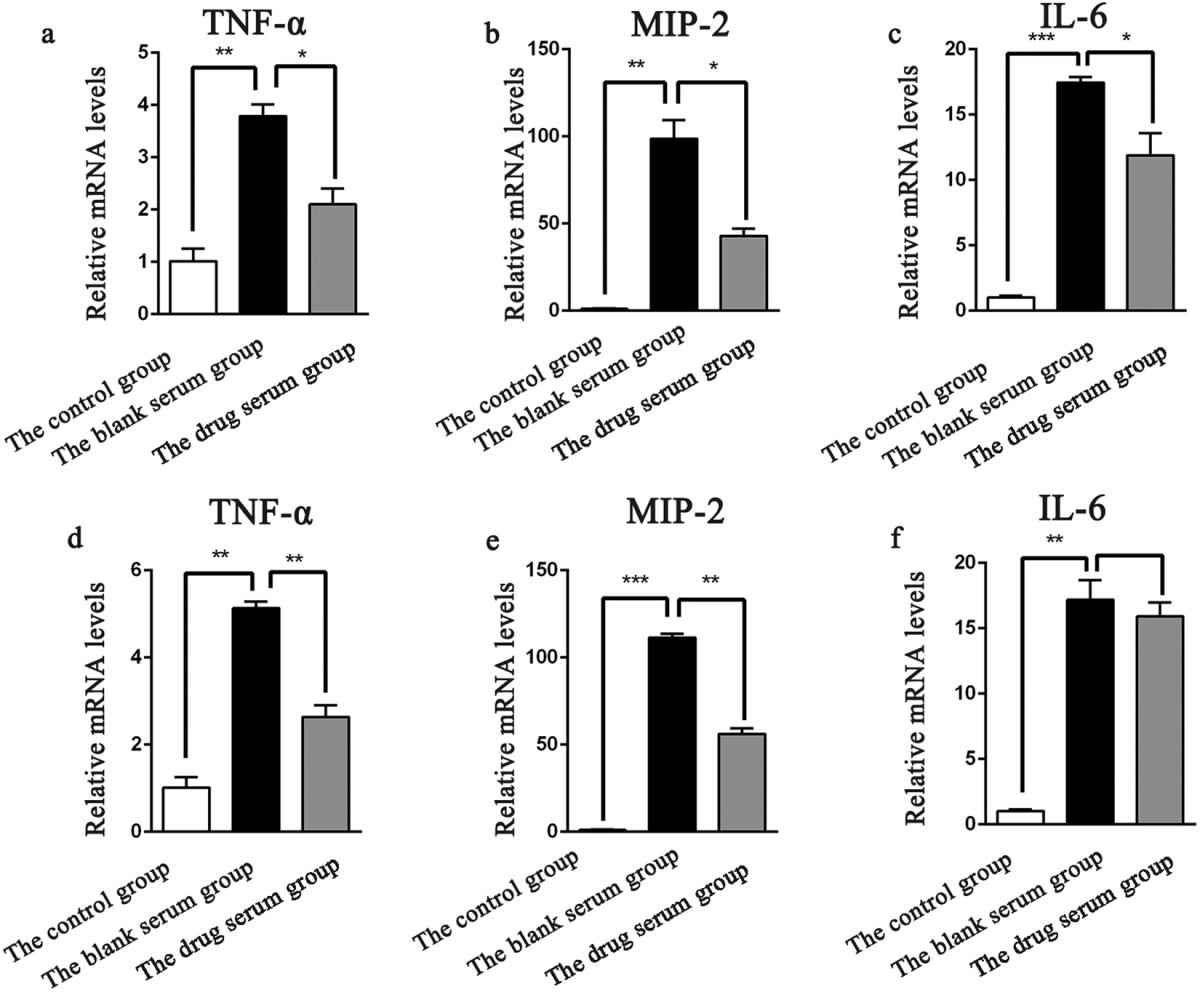
The effect of GFW serum on the mRNA expression levels in Raw264.7 cells. Raw264.7 were pretreated with 10% GFW serum for 24 h.Then the cells were incubated with low concentration (50 ng/ml) or high concentration (100 ng/ml) of bleomycin (BLM) in the presence of lipopolysaccharide (LPS, 100 ng/ml) for 12 h.The expression levels of TNF-α, MIP-2 and IL-6 mRNAs were measured using RT-qPCR. (**a**–**c**) The effects of 10% GFW serum on the expression levels of TNF-α, MIP-2 and IL-6 mRNAs at low concentration of BLM in presence of LPS. (**d**–**f**) The effects of 10% GFW serum on the expression levels of TNF-α, MIP-2 and IL-6 mRNAs at high concentration of BLM in presence of LPS. *P < 0.05, **P < 0.01, ***P < 0.001.

Combining previous formula with PPI enrichment analysis result which was shown in Fig. 7 indicate TLR-4 signaling plays a role in GFW treatment of SSc immune disorders. Growing evidence has confirmed the pivotal role of TLR4-mediated innate immune in SSc. The expression of TLR-4 was found up-regulated in the skin and lung of SSc patients, what’s more, the expression level correlate with the severity of skin disease^75–77^. Upon TLR-4 stimulation induces overexpression of proinflammatory chemokines, macrophage activation and fibrotic responses in SSc^78^. To investigate whether GFW regulate immune disorder of SSc via TLR-4 Signaling, the related up-regulated cytokine such as Interleukin-6 (IL-6), tumor necrosis factor alpha (TNF-α), macrophage inflammatory protein 2 (MIP-2) were examined. The RAW264.7 murine macrophages were pretreated with 10% GFW serum (complete culture medium containing 10% GFW serum) with low concentration (50 ng/ml) or high concentration (100 ng/ml) of bleomycin (BLM) in the presence of lipopolysaccharide (LPS, 100 ng/ml). Expression of TLR4 signaling related cytokines mentioned above were examined using Real-Time Quantitative PCR (RT-qPCR). As shown in Fig. 8, the mRNA expression levels of TNF-α, IL-6, MIP-2 were significantly increased in LPS and BLM induced RAW264.7 macrophages at both concentrations compared to the control. GFW treatment dramatically inhibited the expression of TNF-α, IL-6, MIP-2, but only slightly affected the expression of IL-6 mRNA stimulated with high level BLM. The results suggested GFW take effect in immune disorder may through down-regulation of activated TLR-4 signaling related cytokine expression in RAW264.7 cells.

## Discussion

GFW, a classic TCM prescription, applied to promote blood circulation and thus dissipate blood stasis. It has been widely used in the treatment of SSc with satisfactory clinical effect. However, compared to modern medicine, TCM differ in substance, methodology and philosophy, which hamper its acceptance and recognition in the western biomedical mainstream. A well-understood method to demonstrate the role of GFW in SSc is needed and may facilitate the spread of the formula^79^. Based on this, in this study we tried to elucidate the pharmacological and molecular mechanisms of GFW during the treatment of SSc by the systems biology approach, which could integrate multi-level data for comprehensive analysis. Formula herbs, herb related compounds and targets were gained to get a general view of the GFW. The major property of prescription involves in protecting the vascular endothelium, dilating blood vessels, regulating the immune system and anti-fibrosis according to the main compounds related to the herbs. For example, the total glucosides of paeony (TGP) extracted from the Paeonia albiflora, as an immunomodulatory drug, has been extensively used in autoimmune disease while the compound paeoniflorin can suppress the synthesis of type I collagen to prevent fibrosis progression^80^. To further explore the interactions of compound related targets, we discovered the shared and unique targets take effects in the treatment of SSc coordinately. For example, the shared targets monocyte chemoattractant protein 1 (CCL; MCP-2) and unique target CTGF both mediate pro-fibrotic effects in SSc, CCL2 increases the production of collagen in dermal fibroblasts by inducing the differentiation of interleukin-4 (IL-4)-producing T cells^81^, and CTGF favor ECM accumulation to promote tissue fibrosis^82^. Interfering with multiple targets simultaneously in the same pathological processes, such as the fibrotic procedure can amplify the therapeutic effect. Functional analysis of GFW targets suggested that the therapeutic effect of GFW decoction may be caused by the regulation of HIF-1 signaling pathway, vascular smooth muscle contraction, Toll-like receptor signaling pathway, Biosynthesis of antibiotics, which are related to the characteristic manifestations of scleroderma: tissue fibrosis, microvascular damage, activation of the immune system. We constructed a formula-disease-drug targets network, which showed GFW targets take therapeutic effects by covering 3 representative pathological processes: vascular abnormalities, immune dysregulations and tissue fibrosis. For example, formula targets STAT1 and Smad3 involved in the classic pro-fibrotic processes such as TGF-β signaling pathway, EGFR influenced the vascular smooth muscle cells remodeling and humoral immunity in SSc. It demonstrated that multiple GFW targets participated in different aspects of pathological processes, which confirming the complex pathophysiology of SSc. Therefore, systems biology approach gives a systemic method to evaluate the functions of GFW in the treatment of SSc.

SSc is a complicated disease with elusive pathogenesis. Current therapies focus on the disease related symptoms and involved organs. To maximize their clinical benefits, combined drugs treatment targeting multiple targets and different pathways is recommended. Such treatment mode is similar to Traditional Chinese Medicine treatment of multi-component, multi-target, multi-pathway, multi-link treatment, which has been used in clinical practice for thousands of years^83^. It’s characterized by targeting multiple links of the disease process relatively with the minor side effects. The core concept of TCM is “Holism”, which regard the organism as a whole and whose treatment aim is to restore the overall physiological function. In this study, we tried to predict the molecular mechanism of GFW for treating SSc in a holism view and we concluded that GFW may take effects in 3 pathogenic links of SSc: vascular abnormalities, immune dysregulations and tissue fibrosis. Each pathogenic process involves several distinct pathways to work in coordination. Moreover, in the same aspect, different targets involved in the same signaling pathway play a synergetic role. For example, formula targets ATF3 and Smad3 defined as skin pro-fibrotic determinants both participate in the TGF-β signaling pathway. ATF3 interacts with Smad3 directly on stimulation with TGF-β and regulates Smad activity^84,85^.

In this study, we carried out vitro experiments to validate the above prediction. Previous studies have confirmed the proliferation of pulmonary fibroblasts and production of high amounts collagen contribute to lung fibrosis, which has been regarded as the major cause of death in SSc^86,87^. Therefore, we conducted the CCK8 assay and found GFW can inhibit the proliferation of human lung fibroblast in a dose-effect manner. In addition, the role of GFW on immune disorder was also confirmed by quantitative analysis of TLR-4 related cytokines. The experiment results supported the prediction that GFW can relieve SSc through interfering the section of immune dysregulation and tissue fibrosis.

In summary, combined the application of the systems biology approach with vitro experiments indicate GFW systematically take effects by interfering with the crucial pathogenic links of SSc.

## Materials and Methods

### Data availability and processing

We retrieved 214 herb compositions from TCMID database (http://www.megabionet.org/tcmid/) for further analysis. 1645 high score (>0.7) targets were filtered out according to the recommended confidence range defined by STITCH (low confidence: score <0.4; medium: 0.4 to 0.7; high: > 0.7). SSc associated disease genes came from 3 databases: OMIM (http://www.omim.org), GAD (http://geneticassociationdb.nih.gov/), and CATALOG (http://www.ebi.ac.uk/gwas/). Drug targets were derived from the DrugBank (https://www.drugbank.ca/) according to the recommended medication from expert guidelines^3,5,88^. The relationship among the 3 subsets was visualized in Cytoscape 3.5.0 with ClueGO plug-in. Venn diagram was designed by FunRich software 3.1.3. KEGG pathway enrichment analysis was conducted by online website DAVID Bioinformatics Resources 6.8 (https://david.ncifcrf.gov/), and 136 biological pathways related to SSc were filtered out with adjust P < 0.05.

### Microarray data processing

We obtained 5 expression series matrix (GSE75173, GSE40839, GSE76808, GSE81292, GSE55036) from the Gene Expression Omnibus database (GEO database). Expression values were normalized by RMA function in R. Totally we got 121 microarrays, including 15 normal samples, 61 SSc disease samples and 45 SSc treatment samples (Supplement Table S8). Then the probe ID was transferred into the corresponding genes. “Limma” package was utilized to pick out genes that were differentially expressed among the above 3 types of samples. Genes with fold change value larger than 2 and adjust P-value less than 0.05 were defined as DEGs.

### Target association network and module analysis

The protein-protein interactions collected from BioGRID 3.4 (https://thebiogrid.org/) were performed to construct the disease gene association network. The network was decomposed into 24 modules by MCODE plug-in board of Cytoscape 3.5.0 and Gene ontology (GO) analysis was used for functional annotation of each module.

### Preparation of GFW freeze-dried powder and drug serum

Subsequent to obtaining approval from the Ethics Committee of Zhejiang Chinese Medical University and methods were carried out in accordance with the approved guidelines. All the herbal medicines of GFW, including Cinnamomum cassia (GUI ZHI) 15 g, Poria cocos (FU LING) 15 g, Semen persicae (TAO REN) 15 g, Paeonia albiflora (CHI SHAO) 15 g, Cortex moutan (MU DAN PI) 15 g, were purchased from Chinese Herbal Medicine of Zhejiang Chinese Medical University (Hangzhou, China), which were boiled by water. The filtrates of GFW were combined and concentrated to the crude drug concentration of 1.0 g/ml. Then the filtrates were freeze-dried into power by vacuum freeze dryer (Christ, Germany).

20 male 8-week-old SD rats (300–350 g) were kept in Experimental Animal Center of Zhejiang Chinese Medical University (Hangzhou, China).The rats were randomly divided into two groups, the drug serum group was administered by 0.1 ml/100 g water decoction at crude drug concentration of 2.16 g/ml daily and the blank serum group was given the same volume of physiologic saline for 3 days. Blood was acquired from the rats 1 h after the last time of administration. The serum was collected by centrifugation (3000 rpm for 15 min) and then filtered through a 0.22 μm cellulose acetate membrane.

### CCK8 cell proliferation assay

MRC-5 cells were cultured in 96-well plates at a density of 6 × 10^3^ per well and allowed to attach overnight. Cells were treated with different concentration of GFW freeze-dried powder (0, 50, 250, 625 and 1250 μg/ml) for 48 hours. Then 10 μl of Enhanced Cell Counting Kit-8 solution (CCK8; Beyotime; China) was added to each well. 1 hour later, the OD value was measured at 450 nm using an automatic multiwell spectrophotometer (Thermo Scientific, USA).

### LDH release assay

The cytotoxic effects of GFW freeze-dried powder were measured by LDH Cytotoxicity Assay Kit (Beyotime; China). MRC-5 cells were cultured in 96-well plates at a density of 6 × 10^3^ per well and allowed to attach overnight. The cells were treated with different concentration of GFW freeze-dried powder (0, 50, 250, 625 and 1250 μg/ml) for 48 hours. The cell damage was assessed by the release of LDH from the cells. The manipulation was conducted according to the manufacturer’s protocol. Then OD value was measured at 490 nm with an automatic multiwell spectrophotometer (Thermo Scientific, USA). The cell viability was calculated according to the standard equation. All experiments were 5 replicated.

### RT-qPCR

The total RNA of RAW264.7 cells were extracted using TRIzol Reagent (Invitrogen, USA) and then reverse to cDNA using ReverTra Ace qPCR RT Kit (Toyobo, Osaka, Japan) in a Bio-Rad T100™ Thermal Cycler (Bio-Rad, CA, USA). The cDNA was then subject to real-time PCR using UltraSYBR Mixture (cwbiotech, China) in a Roche LightCycler96 (Roche, Switzerland).The primer sequences were designed according to online website PrimerBank (https://pga.mgh.harvard.edu/primerbank/). All of the experiments were 2 replicated. The relative expression of mRNA was calculated according to the ΔΔCq method.

### Statistical analysis

Data were analyzed using GraphPad Prism 6.0 software. All data is presented as mean ± SD. Difference between two groups was analyzed by Unpaired t test. Difference among multiple groups was performed using One-Way ANOVA followed by Tukey’s post-hoc test, with P < 0.05 taken as statistically significant.

## Electronic supplementary material

Supplementary Information

## References

[1] Van den Hoogen F (2013). classification criteria for systemic sclerosis: an American college of rheumatology/European league against rheumatism collaborative initiative. Ann Rheum Dis.

[2] Pattanaik D, Brown M, Postlethwaite BC, Postlethwaite AE (2015). Pathogenesis of Systemic Sclerosis. Front Immunol.

[3] Kowal-Bielecka O (2009). EULAR recommendations for the treatment of systemic sclerosis: a report from the EULAR Scleroderma Trials and Research group (EUSTAR). Ann Rheum Dis.

[4] Guillevin L (2012). Scleroderma renal crisis: a retrospective multicentre study on 91 patients and 427 controls. Rheumatology (Oxford).

[5] Pellar, R. E. & Pope, J. E. Evidence-based management of systemic sclerosis: Navigating recommendations and guidelines. *Semin Arthritis Rheum*, 10.1016/j.semarthrit.2016.12.003 (2016). 10.1016/j.semarthrit.2016.12.003 28088339

[6] Writing Group of Recommendations of Expert Panel from Chinese Geriatrics Society on the Clinical Use of Compound Danshen Dripping, P. Recommendations on the Clinical Use of Compound Danshen Dripping Pills. *Chin Med J (Engl)* **130**, 972–978, 10.4103/0366-6999.204106 (2017). 10.4103/0366-6999.204106 PMC5407045 28397728

[7] Hu C (2014). Guizhi fuling capsule, an ancient Chinese formula, attenuates endometriosis in rats via induction of apoptosis. Climacteric.

[8] Cao H (2003). The study and application of GUI-ZHI-FU-LING-WAN in Japan. Foreign Med (Chin Med).

[9] Yoshihisa Y (2010). The traditional Japanese formula keishibukuryogan inhibits the production of inflammatory cytokines by dermal endothelial cells. Mediators Inflamm.

[10] Inokawa M, Iguchi K, Kohda H (2006). Thermographic evaluation of the efficacy of Kampo medicines. Hiroshima J Med Sci.

[11] Nagata Y (2012). Effect of keishibukuryogan on endothelial function in patients with at least one component of the diagnostic criteria for metabolic syndrome: a controlled clinical trial with crossover design. Evid Based Complement Alternat Med.

[12] Xue YL, Shi HX, Murad F, Bian K (2011). Vasodilatory effects of cinnamaldehyde and its mechanism of action in the rat aorta. Vasc Health Risk Manag.

[13] Tomita T, Hirayama A, Matsui H, Aoyagi K (2017). Effect of Keishibukuryogan, a Japanese Traditional Kampo Prescription, on Improvement of Microcirculation and Oketsu and Induction of Endothelial Nitric Oxide: A Live Imaging Study. Evid Based Complement Alternat Med.

[14] Liu L (2012). Taoren-Honghua herb pair and its main components promoting blood circulation through influencing on hemorheology, plasma coagulation and platelet aggregation. J Ethnopharmacol.

[15] Jin X (2016). Anti-inflammatory and Anti-oxidative Activities of Paeonol and Its Metabolites Through Blocking MAPK/ERK/p38 Signaling Pathway. Inflammation.

[16] Li LC, Kan LD (2017). Traditional Chinese medicine for pulmonary fibrosis therapy: Progress and future prospects. J Ethnopharmacol.

[17] Pastrello C (2014). Integration, visualization and analysis of human interactome. Biochem Biophys Res Commun.

[18] Zhu X, Gerstein M, Snyder M (2007). Getting connected: analysis and principles of biological networks. Genes Dev.

[19] Xue R (2013). TCMID: Traditional Chinese Medicine integrative database for herb molecular mechanism analysis. Nucleic Acids Res.

[20] Chatr-Aryamontri A (2017). The BioGRID interaction database: 2017 update. Nucleic Acids Res.

[21] Amberger JS, Hamosh A (2017). Searching Online Mendelian Inheritance in Man (OMIM): A Knowledgebase of Human Genes and Genetic Phenotypes. Curr Protoc Bioinformatics.

[22] Huang L, Lv Q, Xie D, Shi T, Wen C (2016). Deciphering the Potential Pharmaceutical Mechanism of Chinese Traditional Medicine (Gui-Zhi-Shao-Yao-Zhi-Mu) on Rheumatoid Arthritis. Sci Rep.

[23] Huang L, Lv Q, Liu F, Shi T, Wen C (2015). A Systems Biology-Based Investigation into the Pharmacological Mechanisms of Sheng-ma-bie-jia-tang Acting on Systemic Lupus Erythematosus by Multi-Level Data Integration. Sci Rep.

[24] Sun L (2016). The essential oil from the twigs of Cinnamomum cassia Presl alleviates pain and inflammation in mice. J Ethnopharmacol.

[25] Sun Y (2014). Biological activities and potential health benefits of polysaccharides from Poria cocos and their derivatives. Int J Biol Macromol.

[26] Yang NY, Liu L, Tao WW, Duan JA, Liu XH, Huang SP (2011). Antithrombotic lipids from Semen Persicae. Nat Prod Res.

[27] Xie P, Cui L, Shan Y, Kang WY (2017). Antithrombotic Effect and Mechanism of Radix Paeoniae Rubra. Biomed Res Int.

[28] He DY, Dai SM (2011). Anti-inflammatory and immunomodulatory effects of paeonia lactiflora pall., a traditional chinese herbal medicine. Front Pharmacol.

[29] Liu MH (2017). Prevention of Bleomycin-Induced Pulmonary Inflammation and Fibrosis in Mice by Paeonol. Front Physiol.

[30] Zhai T (2016). Unique immunomodulatory effect of paeoniflorin on type I and II macrophages activities. J Pharmacol Sci.

[31] Gaillard-Bigot F (2016). Treprostinil iontophoresis improves digital blood flow during local cooling in systemic sclerosis. Fund Clin Pharmacol.

[32] Kim KS (2014). Effects of beta-sitosterol derived from Artemisia capillaris on the activated human hepatic stellate cells and dimethylnitrosamine-induced mouse liver fibrosis. BMC Complement Altern Med.

[33] Awad AB, Smith AJ, Fink CS (2001). Plant sterols regulate rat vascular smooth muscle cell growth and prostacyclin release in culture. Prostaglandins Leukot Essent Fatty Acids.

[34] Barnes TC, Spiller DG, Anderson ME, Edwards SW, Moots RJ (2011). Endothelial activation and apoptosis mediated by neutrophil-dependent interleukin 6 trans-signalling: a novel target for systemic sclerosis?. Ann Rheum Dis.

[35] Desallais L (2014). Targeting IL-6 by both passive or active immunization strategies prevents bleomycin-induced skin fibrosis. Arthritis Res Ther.

[36] Zhou B (2017). MicroRNA-202-3p regulates scleroderma fibrosis by targeting matrix metalloproteinase 1. Biomed Pharmacother.

[37] Lu J (2017). Increased expression of latent TGF-beta-binding protein 4 affects the fibrotic process in scleroderma by TGF-beta/SMAD signaling. Lab Invest.

[38] Steen VD, Medsger TA (2000). Long-term outcomes of scleroderma renal crisis. Ann Intern Med.

[39] Sobanski V, Launay D, Hachulla E, Humbert M (2016). Current Approaches to the Treatment of Systemic-Sclerosis-Associated Pulmonary Arterial Hypertension (SSc-PAH). Curr Rheumatol Rep.

[40] Galie N (2016). 2015 ESC/ERS Guidelines for the diagnosis and treatment of pulmonary hypertension: The Joint Task Force for the Diagnosis and Treatment of Pulmonary Hypertension of the European Society of Cardiology (ESC) and the European Respiratory Society (ERS): Endorsed by: Association for European Paediatric and Congenital Cardiology (AEPC), International Society for Heart and Lung Transplantation (ISHLT). Eur Heart J.

[41] Kontogiorgis C (2015). Studies on the antiplatelet and antithrombotic profile of anti-inflammatory coumarin derivatives. J Enzyme Inhib Med Chem.

[42] Ide M (2017). Transforming growth factor beta-inhibitor Repsox downregulates collagen expression of scleroderma dermal fibroblasts and prevents bleomycin-induced mice skin fibrosis. Exp Dermatol.

[43] Li H, Yang L, Zhang Y, Gao Z (2016). Kaempferol inhibits fibroblast collagen synthesis, proliferation and activation in hypertrophic scar via targeting TGF-beta receptor type I. Biomed Pharmacother.

[44] Usategui A, Criado G, Del Rey MJ, Fare R, Pablos JL (2014). Topical vitamin D analogue calcipotriol reduces skin fibrosis in experimental scleroderma. Arch Dermatol Res.

[45] Terao M (2015). A vitamin D analog inhibits Th2 cytokine- and TGFbeta -induced periostin production in fibroblasts: a potential role for vitamin D in skin sclerosis. Dermatoendocrinol.

[46] Ioannou M (2013). Upregulation of VEGF expression is associated with accumulation of HIF-1alpha in the skin of naive scleroderma patients. Mod Rheumatol.

[47] Deidda M (2017). Distinctive metabolomic fingerprint in scleroderma patients with pulmonary arterial hypertension. Int J Cardiol.

[48] Leask A (2011). Possible strategies for anti-fibrotic drug intervention in scleroderma. J Cell Commun Signal.

[49] Dieude P (2011). NLRP1 influences the systemic sclerosis phenotype: a new clue for the contribution of innate immunity in systemic sclerosis-related fibrosing alveolitis pathogenesis. Ann Rheum Dis.

[50] Riccieri V (2011). Abnormal plasma levels of different angiogenic molecules are associated with different clinical manifestations in patients with systemic sclerosis. Clin Exp Rheumatol.

[51] De Santis M (2016). Nailfold videocapillaroscopy and serum VEGF levels in scleroderma are associated with internal organ involvement. Auto Immun Highlights.

[52] Bhattacharyya S (2013). Toll-like receptor 4 signaling augments transforming growth factor-beta responses: a novel mechanism for maintaining and amplifying fibrosis in scleroderma. Am J Pathol.

[53] Dowson C, Simpson N, Duffy L, O’Reilly S (2017). Innate Immunity in Systemic Sclerosis. Curr Rheumatol Rep.

[54] Liu M (2016). New insights into CD4(+) T cell abnormalities in systemic sclerosis. Cytokine Growth Factor Rev.

[55] Krasimirova E (2017). Treg/Th17 cell balance and phytohaemagglutinin activation of T lymphocytes in peripheral blood of systemic sclerosis patients. World J Exp Med.

[56] Chizzolini C, Dufour AM, Brembilla NC (2018). Is there a role for IL-17 in the pathogenesis of systemic sclerosis?. Immunol Lett.

[57] Bader GD, Hogue CW (2003). An automated method for finding molecular complexes in large protein interaction networks. BMC Bioinformatics.

[58] Lakos G (2004). Targeted disruption of TGF-beta/Smad3 signaling modulates skin fibrosis in a mouse model of scleroderma. Am J Pathol.

[59] Lopez-Isac E (2014). A genome-wide association study follow-up suggests a possible role for PPARG in systemic sclerosis susceptibility. Arthritis Res Ther.

[60] Avouac J (2012). Inhibition of activator protein 1 signaling abrogates transforming growth factor beta-mediated activation of fibroblasts and prevents experimental fibrosis. Arthritis Rheum.

[61] Mallano T (2016). Activating transcription factor 3 regulates canonical TGFbeta signalling in systemic sclerosis. Ann Rheum Dis.

[62] Lindahl GE (2013). Microarray profiling reveals suppressed interferon stimulated gene program in fibroblasts from scleroderma-associated interstitial lung disease. Respir Res.

[63] Odani T (2012). Up-regulated expression of HLA-DRB5 transcripts and high frequency of the HLA-DRB5*01:05 allele in scleroderma patients with interstitial lung disease. Rheumatology (Oxford).

[64] Tsou PS, Sawalha AH (2017). Unfolding the pathogenesis of scleroderma through genomics and epigenomics. J Autoimmun.

[65] Arts MR (2014). Systemic sclerosis immunoglobulin induces growth and a pro-fibrotic state in vascular smooth muscle cells through the epidermal growth factor receptor. PLoS One.

[66] Mayes MD (2012). The genetics of scleroderma: looking into the postgenomic era. Curr Opin Rheumatol.

[67] Jin J, Chou C, Lima M, Zhou D, Zhou X (2014). Systemic Sclerosis is a Complex Disease Associated Mainly with Immune Regulatory and InflammatoryGenes. Open Rheumatol.

[68] Meyringer R (2007). Analysis of gene expression patterns in systemic sclerosis fibroblasts using RNA arbitrarily primed-polymerase chain reaction for differential display. J Rheumatol.

[69] Stahn C, Lowenberg M, Hommes DW, Buttgereit F (2007). Molecular mechanisms of glucocorticoid action and selective glucocorticoid receptor agonists. Mol Cell Endocrinol.

[70] Joseph CG (2014). Association of the autoimmune disease scleroderma with an immunologic response to cancer. Science.

[71] Xu GJ (2016). Systematic autoantigen analysis identifies a distinct subtype of scleroderma with coincident cancer. Proc Natl Acad Sci USA.

[72] Ochoa E (2015). Confirmation of CCR6 as a risk factor for anti-topoisomerase I antibodies in systemic sclerosis. Clin Exp Rheumatol.

[73] Mo C, Zeng Z, Deng Q, Ding Y, Xiao R (2018). Imbalance between T helper 17 and regulatory T cell subsets plays a significant role in the pathogenesis of systemic sclerosis. Biomed Pharmacother.

[74] Asano Y (2017). Recent advances in the treatment of skin involvement in systemic sclerosis. Inflamm Regen.

[75] Bhattacharyya S, Varga J (2015). Emerging roles of innate immune signaling and toll-like receptors in fibrosis and systemic sclerosis. Curr Rheumatol Rep.

[76] Bhattacharyya, S. *et al*. TLR4-dependent fibroblast activation drives persistent organ fibrosis in skin and lung. *JCI Insight* **3**, 10.1172/jci.insight.98850 (2018). 10.1172/jci.insight.98850 PMC6124522 29997297

[77] Bhattacharyya S, Varga J (2018). Endogenous ligands of TLR4 promote unresolving tissue fibrosis: Implications for systemic sclerosis and its targeted therapy. Immunol Lett.

[78] Stifano G (2014). Chronic Toll-like receptor 4 stimulation in skin induces inflammation, macrophage activation, transforming growth factor beta signature gene expression, and fibrosis. Arthritis Res Ther.

[79] Cheung F (2011). TCM: Made in China. Nature.

[80] Ji Y (2016). Paeoniflorin suppresses TGF-beta mediated epithelial-mesenchymal transition in pulmonary fibrosis through a Smad-dependent pathway. Acta Pharmacol Sin.

[81] Distler JH (2006). Monocyte chemoattractant protein 1 released from glycosaminoglycans mediates its profibrotic effects in systemic sclerosis via the release of interleukin-4 from T cells. Arthritis Rheum.

[82] Abraham D (2008). Connective tissue growth factor: growth factor, matricellular organizer, fibrotic biomarker or molecular target for anti-fibrotic therapy in SSc?. Rheumatology (Oxford).

[83] Cerinic Matucci M (2007). Therapeutic challenges for systemic sclerosis: facts and future targets. Ann N Y Acad Sci.

[84] Young A, Khanna D (2015). Systemic sclerosis: a systematic review on therapeutic management from 2011 to 2014. Curr Opin Rheumatol.

[85] Yanaba K (2016). Strategy for treatment of fibrosis in systemic sclerosis: Present and future. J Dermatol.

[86] Lei L (2016). Th17 cells and IL-17 promote the skin and lung inflammation and fibrosis process in a bleomycin-induced murine model of systemic sclerosis. Clin Exp Rheumatol.

[87] Hua-Huy T (2010). Increased alveolar concentration of nitric oxide is related to serum-induced lung fibroblast proliferation in patients with systemic sclerosis. J Rheumatol.

[88] Denton CP (2016). BSR and BHPR guideline for the treatment of systemic sclerosis. Rheumatology (Oxford).

[89] Kanehisa M, Furumichi M, Tanabe M, Sato Y, Morishima K (2017). KEGG: new perspectives on genomes, pathways, diseases and drugs. Nucleic Acids Res.

