# Supplementary material for: Deciphering the Potential Pharmaceutical Mechanism of GUI-ZHI-FU-LING-WAN on Systemic Sclerosis based on Systems Biology Approaches
Source: Sci Rep. 2019 Jan 23;9:355. doi: 10.1038/s41598-018-36314-2 (PMC6344516; doi:10.1038/s41598-018-36314-2)

**Deciphering the Potential Pharmaceutical Mechanism of GUI-ZHI-FU-LING-WAN on Systemic Sclerosis based on Systems Biology Approaches**

Qiao Wang^1, #^, Guoshan Shi^2^, Yun Zhang^1^, Feilong Lu^1^, Duoli Xie^1^, Chengping Wen^1, *^, Lin Huang^1, *^

^1^ TCM Clinical Basis Institute, Zhejiang Chinese Medicine University, 548 Binwen Road, Hangzhou, Zhejiang, 310000, China

^2^ Department of Integrative Traditional & Western Medicine, Medical College, Yangzhou University, Yangzhou, Jiangsu 225001, China.

* Correspondence should be addressed to L.H. (E-mail: robinsoina@sina.com Tel: +86-571-86613644.) and C.W. (E-mail: [wengcp@163.com](mailto:wengcp@163.com); Tel: +86-571-86633131.)

**Table S1** Description of the number of herbs and related compounds of GFW formula. 5 herbs and 227 compounds related to GFW were retrieved from TCMID database.

| Herb | Compound |
| --- | --- |
| Cinnamomum cassia | β-sitosterol |
| Cinnamomum cassia | trans-cinnamic acid |
| Cinnamomum cassia | tetradecanal |
| Cinnamomum cassia | styrene |
| Cinnamomum cassia | protocatechuic acid-3-glucoside |
| Cinnamomum cassia | protocatechuic acid |
| Cinnamomum cassia | procurcumenol |
| Cinnamomum cassia | proanthocyanidin b2 |
| Cinnamomum cassia | nerolidol |
| Cinnamomum cassia | melilotocarpan a |
| Cinnamomum cassia | meliloticacid |
| Cinnamomum cassia | gamma-sitosterol |
| Cinnamomum cassia | farnesol |
| Cinnamomum cassia | dihydromelilotoside |
| Cinnamomum cassia | coumarinic acid |
| Cinnamomum cassia | coumarin |
| Cinnamomum cassia | cinnamicalcohol |
| Cinnamomum cassia | cinnamicacid |
| Cinnamomum cassia | cinnamic aldehyde |
| Cinnamomum cassia | cinnamaldehyde |
| Cinnamomum cassia | camphor |
| Cinnamomum cassia | beta-sitoterol |
| Cinnamomum cassia | benzaldehyde |
| Cinnamomum cassia | anhydrocinnzeylanol |
| Cinnamomum cassia | anhydrocinnzeylanine |
| Cinnamomum cassia | anethole |
| Cinnamomum cassia | acetic acid |
| Cinnamomum cassia | 5-cinnamoyl-9-o-acetylphototaxicin i |
| Cinnamomum cassia | 3-o-p-hydroxy-trans-cinnamoylmaslinic acid |
| Cinnamomum cassia | 3,4-dihydroxybenzoicacid |
| Cinnamomum cassia | 3-(4-hdroxyphenyl)-trans-propenoic acid-2,3-dihydroxypropyl ester |
| Cinnamomum cassia | 2-methoxycinnamaldehyde |
| Cinnamomum cassia | 2'-hydroxycinnamaldehyde |
| Cinnamomum cassia | (+-)-2,3-dihydro-2-(1-methylethenyl)-5-benzofurancarboxylic acid methyl ester |
| Poria cocos | 10-hydroxyacetylbaccatin vi |
| Poria cocos | 20-hexadecanoylingenol |
| Poria cocos | 25-hydroxy-3-epidehydrotumulosicacid |
| Poria cocos | 3-epidehydrotumulosicacid |
| Poria cocos | 3β-hydroxy-16α-acetoxy-lanosta-7,9(11),24-trien-21-oicacid |
| Poria cocos | 3β-hydroxylanosta-7,9(11),24-trien-21-oicacid |
| Poria cocos | 3β-p-hydroxybenzoyldehydrotumulosicacid |
| Poria cocos | adenine |
| Poria cocos | beta-amyrin acetate |
| Poria cocos | caprylicacid |
| Poria cocos | capsaicin(e) |
| Poria cocos | choline |
| Poria cocos | chrysanthemaxanthin |
| Poria cocos | cis-4-dodecenoic acid |
| Poria cocos | dehydroabieticacid methyl ester |
| Poria cocos | dehydroeburicoicacid |
| Poria cocos | dehydrotumulosicacid |
| Poria cocos | dodecenoicacid |
| Poria cocos | eburicoicacid |
| Poria cocos | eburicol |
| Poria cocos | ergosterol |
| Poria cocos | ergotamine |
| Poria cocos | hexadecanoicacid |
| Poria cocos | hydrangeic acid |
| Poria cocos | lauric aldehyde |
| Poria cocos | lauricacid |
| Poria cocos | o-acetylpachymic acid-25-ol |
| Poria cocos | pachyman |
| Poria cocos | pachymic acid |
| Poria cocos | pachymicacid methyl ester |
| Poria cocos | pachypodol |
| Poria cocos | p-hydroxybenzyl alcohol |
| Poria cocos | pinicolicacid a |
| Poria cocos | pinidine |
| Poria cocos | polyporenicacid c |
| Poria cocos | polystachoside |
| Poria cocos | poricoic acid b |
| Poria cocos | poricoicacid a |
| Poria cocos | poricoicacid c |
| Poria cocos | poricoicacid d |
| Poria cocos | poricoicacid dm |
| Poria cocos | poricoicacid g |
| Poria cocos | poricoicacid h |
| Poria cocos | porphyroxine |
| Poria cocos | trametenolicacid |
| Poria cocos | tumulosicacid |
| Poria cocos | tumulosicacid methyl ester |
| Poria cocos | turanose |
| Poria cocos | undecan-2-ol |
| Poria cocos | undecanoicacid |
| Poria cocos | β-amyrin acetate |
| Cortex Moutan | sugiol |
| Cortex Moutan | suffruticosol b |
| Cortex Moutan | suffruticosol a |
| Cortex Moutan | suffruticoside d |
| Cortex Moutan | suffruticoside c |
| Cortex Moutan | suffruticoside b |
| Cortex Moutan | suffruticoside a |
| Cortex Moutan | paeonoside |
| Cortex Moutan | paeonolide |
| Cortex Moutan | paeonol |
| Cortex Moutan | paeonilactone a |
| Cortex Moutan | paeoniflorin |
| Cortex Moutan | oxypeucedanin |
| Cortex Moutan | oxypaeoniflorin |
| Cortex Moutan | gallic acid |
| Cortex Moutan | gallic acid-3-o-(6'-o-galloyl)-glucoside |
| Cortex Moutan | benzoylpaeoniflorin |
| Cortex Moutan | benzoyloxypaeoniflorin |
| Cortex Moutan | aplopaeonoside |
| Cortex Moutan | 1,2,3,4,6-pentagalloylglucose |
| Cortex Moutan | (+)-3,3',5',5,7-pentahydroflavanone |
| Semen Persicae | β-sitosterol |
| Semen Persicae | β-daucosterol |
| Semen Persicae | vanilloloside |
| Semen Persicae | uridine |
| Semen Persicae | sucrose |
| Semen Persicae | sambunigrin |
| Semen Persicae | R-mandelamide |
| Semen Persicae | Prupersin E |
| Semen Persicae | Prupersin D |
| Semen Persicae | Prupersin C |
| Semen Persicae | Prupersin B |
| Semen Persicae | Prupersin A |
| Semen Persicae | Prupersin |
| Semen Persicae | oleic acid |
| Semen Persicae | methyl-α-d-fructofuranoside |
| Semen Persicae | linoleic acid |
| Semen Persicae | glycerolandrosin |
| Semen Persicae | ethyl amygdalinate |
| Semen Persicae | ethyl a-D-fructofuranoside |
| Semen Persicae | ergosterol peroxide |
| Semen Persicae | enthyl P-D-glucopyranoside |
| Semen Persicae | dihydrodehydrodiconiferyl alcoh-ol 4-O-P-D-glucopyranosides |
| Semen Persicae | cordycepin |
| Semen Persicae | Benzyl-β-D-glucopyranoside |
| Semen Persicae | benzyl-p-D-glucopyranosyl(1→6)-β-D-glucopyranoside |
| Semen Persicae | benzoic acid 4-O-P-D-glucopyranoside |
| Semen Persicae | amygdalin |
| Semen Persicae | alpha-methylfurfural |
| Semen Persicae | adenosine |
| Semen Persicae | 4-hydroxymethyl-2-methoxyphenyl 6-O-benzoyl-β-D-glucopyranoside |
| Semen Persicae | 2-p-D-glucopyranos-yloxy-2-phenylacetic acid amide |
| Semen Persicae | 1-O-vanilloyl-β-D-glucoside |
| Semen Persicae | (7R)-mandelic acid 7-O-β-D-glucopyranoside |
| Semen Persicae | (+)-catechin |
| Semen Persicae | (-)-secoisoIariciresinol 4-O-p-D-glucopyranoside |
| Paeonia albiflora | β-sitosterol-α- glucoside |
| Paeonia albiflora | z-1s,5R-β-pinen-10-yl-β-vicianoside |
| Paeonia albiflora | vanillic acid |
| Paeonia albiflora | propyl gallate |
| Paeonia albiflora | pedunculagin |
| Paeonia albiflora | palmicacid |
| Paeonia albiflora | palbinone |
| Paeonia albiflora | pailactone B |
| Paeonia albiflora | paeonoside |
| Paeonia albiflora | paeonolide |
| Paeonia albiflora | paeonol |
| Paeonia albiflora | paeonin |
| Paeonia albiflora | paeonilactone c |
| Paeonia albiflora | paeonilactone b |
| Paeonia albiflora | paeonilactone a |
| Paeonia albiflora | paeonilactinone |
| Paeonia albiflora | paeoniflorin |
| Paeonia albiflora | paeoniflorigenone |
| Paeonia albiflora | paeonflorin |
| Paeonia albiflora | oxypaeoniflorin |
| Paeonia albiflora | oxypaeoniflora |
| Paeonia albiflora | oleanolic acid |
| Paeonia albiflora | naringenin |
| Paeonia albiflora | methyl gallate |
| Paeonia albiflora | lactinolide |
| Paeonia albiflora | lactiflorin |
| Paeonia albiflora | kaempferol-3-O-β-D-glucoside |
| Paeonia albiflora | kaempferol-3,7-di-O-β-D-glucoside |
| Paeonia albiflora | kaempferol |
| Paeonia albiflora | hederagenin |
| Paeonia albiflora | galloylpaeoniflorin |
| Paeonia albiflora | gallotannin |
| Paeonia albiflora | gallotannic acid |
| Paeonia albiflora | gallocatechin |
| Paeonia albiflora | gallic acid |
| Paeonia albiflora | friedelin |
| Paeonia albiflora | evofo lin B |
| Paeonia albiflora | eugeniin |
| Paeonia albiflora | ethyl gallate |
| Paeonia albiflora | epigallocatechin |
| Paeonia albiflora | epifriedelanol |
| Paeonia albiflora | epi-4-ethyl-benzoyl-paeoniflorin |
| Paeonia albiflora | ellagitannin |
| Paeonia albiflora | dihydroquercetin |
| Paeonia albiflora | dihydrokaempferol |
| Paeonia albiflora | d-catechin |
| Paeonia albiflora | daucosterol |
| Paeonia albiflora | catechin |
| Paeonia albiflora | butyl gallate |
| Paeonia albiflora | betulinic acid |
| Paeonia albiflora | beta-sitosterol |
| Paeonia albiflora | benzoylpaeoniflorin |
| Paeonia albiflora | benzoyloxypaeoniflorin |
| Paeonia albiflora | benzoate |
| Paeonia albiflora | arbutin |
| Paeonia albiflora | albiflorin |
| Paeonia albiflora | acioflorin |
| Paeonia albiflora | acetic acid |
| Paeonia albiflora | 9-ethyl-neopaeoniaflorin A |
| Paeonia albiflora | 6-O-β-D-gluecopyranosyllactinolid |
| Paeonia albiflora | 6′-O-benzoyl-4″-hydroxy-3″-methoxy-paeoniflorin |
| Paeonia albiflora | 4-ethyl-paeoniflorin |
| Paeonia albiflora | 4-ethyl-benzoyl-paeoniflorin |
| Paeonia albiflora | 3β-hydroxyolean-12-en-28-al |
| Paeonia albiflora | 3β-hydroxy-11α,12α-epoxy-olean-28,13β-olide |
| Paeonia albiflora | 3β-hydroxy-11-oxo-olean-12-en-28-oic acid |
| Paeonia albiflora | 30-norhederagenin |
| Paeonia albiflora | 3,4′-O-dimethylellagic acid |
| Paeonia albiflora | 3,4,4′-O-trimethyl-5-hydroxy-5-methyl ellagic acid |
| Paeonia albiflora | 3,3′-O-dimethyl ellagic acid |
| Paeonia albiflora | 3,3′,4-O-trimethyl ellagic acid |
| Paeonia albiflora | 23β-hydroxyolean-12-en-28-al |
| Paeonia albiflora | 23-hydroxy betulinic acid |
| Paeonia albiflora | 1-O-β-D-gluecopyranosyl-paeonisuffrone |
| Paeonia albiflora | 1-O-galloylglucose |
| Paeonia albiflora | 11α,12α-epoxy-3β,23-dihydroxyolean-28,13β-olide |
| Paeonia albiflora | 11α,12α-epoxy-3β,23-dihydroxy-30-norolean-20-en-28,13β-olide |
| Paeonia albiflora | 1,2,3,6-pentagalloylglucose |
| Paeonia albiflora | 1,2,3,4,6-pentagalloylglucose |
| Paeonia albiflora | 1,2,3,4,6-O-pentagalloylglucose |
| Paeonia albiflora | (Z)-(1S,5R)-β-pinen-10-yl-β-vicianoside |
| Paeonia albiflora | (2)-(1s, 5r)-beta-pinene-10-y1-beta-vicianoside |
| Paeonia albiflora | (1S,2S,4R)-trans-2-hydroxy-1,8-cineole-β-D-glucopyranoside |
| Paeonia albiflora | (1R,2S,4R)-trans-1,8-cineole-2-O-β-Dglucopyranoside |
| Paeonia albiflora | (+)-catechin |
| Paeonia albiflora | (-)-catechin |

Table S2 The list of the shared targets of GFW compounds with the frequency in descending order.

| **Compounds** | **Shared targets** | **Frequency** |
| --- | --- | --- |
| amygdalin;arbutin | BDKRB1 | 170 |
| amygdalin;camphor | BDKRB1 | 170 |
| arbutin;camphor | BDKRB1 | 170 |
| arbutin;coumarin | NMUR1 | 170 |
| camphor;coumarin | NMUR1;BDKRB1 | 170 |
| adenosine;coumarin | NMUR1 | 164 |
| amygdalin;coumarin | NMUR1 | 160 |
| adenosine;arbutin | BDKRB1 | 154 |
| adenosine;amygdalin | BDKRB1 | 152 |
| adenosine;camphor | BDKRB1 | 152 |
| acetic acid;oleic acid | NMUR1 | 123 |
| acetic acid;lauricacid | QRFP;SAA1;NPSR1;AVPR1A;EDN1;GCGR;GHSR;KISS1R;LTB4R;MLNR;NTSR1;OPN4;NTSR2;CYSLTR1;EDN2;EDN3;GM2A;GNA11;GNA14;GNAQ;GPR132;MLN;NTS;TACR2;NMUR1 | 107 |
| amygdalin;ergotamine | BDKRB1 | 55 |
| arbutin;ergotamine | BDKRB1 | 55 |
| camphor;ergotamine | BDKRB1 | 55 |
| coumarin;ergotamine | BDKRB1 | 55 |
| adenosine;ergotamine | CASP3 | 52 |
| oleic acid;ergotamine | CASP3 | 47 |
| oleic acid;linoleic acid | ACOT1;ACOT2;ACOT4;ACOT7;CYP4A11;FABP4;ACSM1;RPE65;SLC27A1 | 47 |
| acetic acid;adenosine | NMUR1;PMCH;ANXA1;APP;CASR;FPR2;GNG2;GPR17;KNG1;LPAR5;MTRNR2L12;NMS;NMU;GCG;FOS;NPSR1;AGT;EDN1;GCGR;HCRT;NOS1;NPS;OXT;MMP9;PNP;TP53;VEGFA;AURKA;CFTR;FYN;HINT1;HINT2;PIM1;PRKACA;RNASE1;SAA1;AVP;REN;NOS3;SRPK1;SRPK2;ADK;DDC;MAT2A;NTRK1;RDH14 | 46 |
| lauricacid;ergotamine | BDKRB1 | 46 |
| acetic acid;linoleic acid | FOS | 35 |
| adenosine;oleic acid | BDKRB1 | 29 |
| acetic acid;ergotamine | NMUR1;LPAR1;AGTR1;NPSR1;AVPR1A;CCKAR;EDNRB;GHSR;GNRHR;GPR65;GRPR;HCRTR1;HCRTR2;HTR2A;KISS1R;LTB4R;LTB4R2;MLNR;NTSR1;NTSR2;OPN4;PROKR1;PROKR2;PTGFR;QRFPR;TBXA2R;TRHR;RHO | 28 |
| adenosine;lauricacid | NMUR1 | 28 |
| lauricacid;oleic acid | QRFP;BDKRB1 | 28 |
| coumarin;lauricacid | BDKRB1 | 23 |
| amygdalin;lauricacid | NMUR1 | 22 |
| amygdalin;oleic acid | BDKRB1 | 22 |
| arbutin;lauricacid | NMUR1 | 22 |
| arbutin;oleic acid | BDKRB1 | 22 |
| camphor;lauricacid | NMUR1 | 22 |
| camphor;oleic acid | BDKRB1 | 22 |
| acetic acid;sucrose | PMCH | 19 |
| coumarin;oleic acid | BDKRB1 | 19 |
| acetic acid;coumarin | NMUR1;PMCH;ANXA1;APP;CASR;FPR2;GNG2;GPR17;KNG1;LPAR1;LPAR5;MTRNR2L12;NMS;NMU;AGT;CA2;SAA1 | 17 |
| acetic acid;amygdalin | NMUR1;PMCH;ANXA1;APP;CASR;FPR2;GNG2;GPR17;KNG1;LPAR1;LPAR5;MTRNR2L12;NMS;NMU;AGT;SAA1 | 16 |
| acetic acid;arbutin | NMUR1;PMCH;ANXA1;APP;CASR;FPR2;GNG2;GPR17;KNG1;LPAR1;LPAR5;MTRNR2L12;NMS;NMU;AGT;SAA1 | 16 |
| acetic acid;camphor | NMUR1;PMCH;ANXA1;APP;CASR;FPR2;GNG2;GPR17;KNG1;LPAR1;LPAR5;MTRNR2L12;NMS;NMU;AGT;SAA1 | 16 |
| acetic acid;uridine | P2RY6 | 13 |
| adenosine;capsaicin(e) | IL8 | 13 |
| adenosine;uridine | SLC29A2 | 12 |
| acetic acid;adenine | P2RY1;PNP;APRT;HPRT1;HSP90AA1;PECR;SRPK1;SRPK2;ALKBH5;ALKBH8;MTAP | 11 |
| acetic acid;capsaicin(e) | FOS;CCK;EDN1;GAST;PPARA;TACR1;SRC;TP53;CBS;GCG;VEGFA | 11 |
| lauricacid;linoleic acid | AGT | 11 |
| acetic acid;choline | GCG;LYZ;ACHE;BCHE;CHDH;S100A4;REN;ADH4;ADH7 | 9 |
| cinnamaldehyde;cinnamic aldehyde | CASP3;PTGS2;CASP8;SLC2A4;MAPK14;MAPK8;MAPK1;MAPK3;NOS2 | 9 |
| adenosine;sucrose | PMCH | 8 |
| caprylicacid;linoleic acid | GLTP;PAEP;ACSL6;ACSBG1;ACSL1;ACSL3;ACSL4;ACSL5 | 8 |
| caprylicacid;oleic acid | GLTP;PAEP;ACSL1;ACSL3;ACSL4;ACSL5;ACSL6 | 7 |
| capsaicin(e);lauricacid | BDKRB1;PTGS2;GCG;CCK;EDN1;GAST;TACR1 | 7 |
| capsaicin(e);oleic acid | CASP3;BDKRB1;GCG;CCK;GAST;TACR1;STAT3 | 7 |
| acetic acid;kaempferol | NOS1;ESR2;SRC;CCL2;CDK2;GSTP1 | 6 |
| adenine;adenosine | PNP | 6 |
| capsaicin(e);cinnamaldehyde | CASP3;PTGS2;CASP8;MAPK8;MAPK1;MAPK3 | 6 |
| capsaicin(e);cinnamic aldehyde | CASP3;PTGS2;CASP8;MAPK8;MAPK1;MAPK4 | 6 |
| capsaicin(e);linoleic acid | FOS;IL6;MAPK8;PPARA;CYP1A2;CYP2D6 | 6 |
| acetic acid;protocatechuic acid | MPO | 5 |
| adenosine;cordycepin | ADORA1 | 5 |
| adenosine;linoleic acid | FOS | 5 |
| camphor;capsaicin(e) | IL8;GAL;POMC;TRPV1;BDKRB1 | 5 |
| capsaicin(e);coumarin | BDKRB1;PARP1;IL8;GAL;POMC | 5 |
| capsaicin(e);oleanolic acid | CASP3;CASP8;CYP1A2;CYP3A4;PPARA | 5 |
| acetic acid;cordycepin | NOS1;MMP9;LEPR;FOXP3 | 4 |
| amygdalin;capsaicin(e) | IL8;GAL;POMC;BDKRB1 | 4 |
| arbutin;capsaicin（e） | IL8 | 4 |
| 1,2,3,4,6-O-pentagalloylglucose;1,2,3,4,6-pentagalloylglucose | HMOX1;TMPRSS11D;ESR1;EGFR | 3 |
| acetic acid;3,4-dihydroxybenzoicacid | MPO;DPYD;NR5A1 | 3 |
| acetic acid;gallic acid | MPO;EIF2AK3;SERPINE1 | 3 |
| adenosine;choline | GCG | 3 |
| adenosine;kaempferol | HMOX1 | 3 |
| adenosine;oleanolic acid | CASP3 | 3 |
| betulinic acid;oleanolic acid | CASP3;TOP2A;TOP1 | 3 |
| capsaicin(e);cordycepin | IL6;CASP9;TMPRSS11D | 3 |
| capsaicin(e);gallic acid | CASP3;CASP7;ATM | 3 |
| choline;oleic acid | BDNF;CHAT;GCG | 3 |
| coumarin;loleic acid | LPAR3;MCHR2;NMUR2 | 3 |
| coumarin;sucrose | PMCH;LTF;TF | 3 |
| lauricacid;sucrose | PMCH;AGTR1;LTF | 3 |
| oleic acid;β-sitosterol | CASP3;APOE;ICAM1 | 3 |
| 3,4-dihydroxybenzoicacid;protocatechuic acid | COMT;DHODH | 2 |
| 3,5-dihydroxybenzoicacid;acetic acid | COMT;DHODH | 2 |
| acetic acid;caprylicacid | ALDH3A2;ALDH9A1 | 2 |
| acetic acid;cinnamaldehyde | MAPK14;NOS2 | 2 |
| acetic acid;cinnamic aldehyde | MAPK14;NOS2 | 2 |
| acetic acid;oleanolic acid | PPARA | 2 |
| adenine;uridine | SLC29A2 | 2 |
| adenosine;betulinic acid | CASP3 | 2 |
| adenosine;cinnamaldehyde | CASP3 | 2 |
| adenosine;cinnamic aldehyde | CASP3 | 2 |
| adenosine;gallic acid | CASP3 | 2 |
| arbutin;linoleic acid | TYR;AGT | 2 |
| betulinic acid;capsaicin(e) | CASP7;CASP3 | 2 |
| betulinic acid;gallic acid | CASP3;CASP7 | 2 |
| caprylicacid;lauricacid | GLTP;PAEP | 2 |
| capsaicin(e);ergotamine | CASP3;BDKRB1 | 2 |
| capsaicin(e);kaempferol | IL2;STAT1;SRC;STAT3 | 2 |
| capsaicin(e);paeonol | PTGS2;CASP8 | 2 |
| choline;lauricacid | ADH5;GCG | 2 |
| choline;sucrose | REN;LYZ | 2 |
| cinnamaldehyde;oleanolic acid | CASP3;CASP8 | 2 |
| cinnamaldehyde;paeonol | PTGS2;CASP8 | 2 |
| cinnamic aldehyde;oleanolic acid | CASP3;CASP8 | 2 |
| cinnamic aldehyde;paeonol | PTGS2;CASP8 | 2 |
| cinnamicacid;trans-cinnamic acid | ADIPOQ;KDM1A | 2 |
| ergotamine;oleanolic acid | CASP3;PTGIR | 2 |
| ergotamine;sucrose | AGTR1;ADRB2 | 2 |
| gallic acid;kaempferol | JUN;AKT1 | 2 |
| gallic acid;linoleic acid | TYR;SERPINE1 | 2 |
| kaempferol;sucrose | CYP2B6;HMOX1 | 2 |
| lauricacid;adenine | PTGS2;P2RY1 | 2 |
| linoleic acid;oleanolic acid | PPARA;CYP1A2 | 2 |
| linoleic acid;paeonol | TYR;VCAM1 | 2 |
| linoleic acid;β-sitosterol | APOE;SREBF2 | 2 |
| oleic acid;sucrose | PMCH;AGTR1 | 2 |
| paeonol;adenosine | CASP8;TYR | 2 |

Table S3 KEGG pathway enrichment analysis of GFW formula. 1645 GFW formula targets were performed the pathway enrichment analysis by online website DAVID Bioinformatics Resources 6.8 (<https://david.ncifcrf.gov/>), 136 pathways were filtered according to P-value ≤ 0.05.

| Category | P-Value | Category | P-Value |
| --- | --- | --- | --- |
| Neuroactive ligand-receptor interaction | 1.40E-78 | Dopaminergic synapse | 2.40E-04 |
| Metabolic pathways | 1.20E-23 | Regulation of lipolysis in adipocytes | 3.40E-04 |
| Calcium signaling pathway | 2.20E-18 | Renin-angiotensin system | 4.30E-04 |
| Linoleic acid metabolism | 2.20E-16 | Malaria | 4.50E-04 |
| Metabolism of xenobiotics by cytochrome P450 | 2.20E-15 | Rheumatoid arthritis | 5.90E-04 |
| Taste transduction | 5.40E-14 | Pyruvate metabolism | 1.00E-03 |
| Arachidonic acid metabolism | 8.50E-14 | Thyroid cancer | 1.20E-03 |
| PPAR signaling pathway | 2.10E-12 | Ovarian steroidogenesis | 1.30E-03 |
| Chemical carcinogenesis | 2.70E-12 | Cholinergic synapse | 1.40E-03 |
| Serotonergic synapse | 3.60E-12 | Osteoclast differentiation | 1.70E-03 |
| cAMP signaling pathway | 3.50E-11 | Purine metabolism | 2.00E-03 |
| Ether lipid metabolism | 3.00E-10 | Fc epsilon RI signaling pathway | 2.10E-03 |
| Chemokine signaling pathway | 3.20E-10 | Alzheimer's disease | 2.50E-03 |
| Drug metabolism - cytochrome P450 | 6.10E-10 | B cell receptor signaling pathway | 2.60E-03 |
| Retinol metabolism | 7.00E-10 | Toll-like receptor signaling pathway | 2.80E-03 |
| alpha-Linolenic acid metabolism | 9.70E-10 | Proteoglycans in cancer | 3.10E-03 |
| Adipocytokine signaling pathway | 1.60E-09 | Steroid hormone biosynthesis | 4.00E-03 |
| Pyrimidine metabolism | 3.60E-09 | Measles | 4.20E-03 |
| Long-term depression | 3.60E-08 | Prion diseases | 4.30E-03 |
| Fat digestion and absorption | 8.40E-08 | Porphyrin and chlorophyll metabolism | 5.30E-03 |
| Vascular smooth muscle contraction | 1.10E-07 | NOD-like receptor signaling pathway | 5.40E-03 |
| Sphingolipid signaling pathway | 1.50E-07 | Terpenoid backbone biosynthesis | 5.40E-03 |
| Renin secretion | 2.00E-07 | Non-alcoholic fatty liver disease (NAFLD) | 6.20E-03 |
| Pertussis | 2.20E-07 | Intestinal immune network for IgA production | 6.30E-03 |
| Chagas disease (American trypanosomiasis) | 4.40E-07 | Non-small cell lung cancer | 6.60E-03 |
| Choline metabolism in cancer | 5.80E-07 | AMPK signaling pathway | 6.70E-03 |
| TNF signaling pathway | 7.70E-07 | Oxytocin signaling pathway | 7.70E-03 |
| GnRH signaling pathway | 9.80E-07 | Insulin signaling pathway | 7.70E-03 |
| VEGF signaling pathway | 1.00E-06 | Butanoate metabolism | 7.80E-03 |
| Insulin resistance | 1.30E-06 | Glyoxylate and dicarboxylate metabolism | 7.80E-03 |
| Inflammatory mediator regulation of TRP channels | 2.30E-06 | Viral myocarditis | 8.00E-03 |
| Legionellosis | 4.30E-06 | Apoptosis | 8.70E-03 |
| Pathways in cancer | 5.10E-06 | Colorectal cancer | 8.70E-03 |
| Fatty acid degradation | 5.50E-06 | Circadian entrainment | 8.90E-03 |
| Long-term potentiation | 6.00E-06 | Pentose and glucuronate interconversions | 9.50E-03 |
| Cytokine-cytokine receptor interaction | 6.00E-06 | Prostate cancer | 1.30E-02 |
| Tuberculosis | 8.60E-06 | Complement and coagulation cascades | 1.30E-02 |
| Histidine metabolism | 1.30E-05 | Ras signaling pathway | 1.40E-02 |
| Amyotrophic lateral sclerosis (ALS) | 1.50E-05 | Glutathione metabolism | 1.40E-02 |
| Pancreatic secretion | 1.50E-05 | Glioma | 1.40E-02 |
| Estrogen signaling pathway | 2.40E-05 | Acute myeloid leukemia | 1.50E-02 |
| Glycerophospholipid metabolism | 2.50E-05 | Valine, leucine and isoleucine degradation | 1.50E-02 |
| Bladder cancer | 2.90E-05 | Endometrial cancer | 1.70E-02 |
| Fatty acid biosynthesis | 3.10E-05 | Melanogenesis | 1.70E-02 |
| Gap junction | 3.40E-05 | Adherens junction | 1.80E-02 |
| Cocaine addiction | 3.90E-05 | Amoebiasis | 1.90E-02 |
| Glutamatergic synapse | 3.90E-05 | Chronic myeloid leukemia | 2.00E-02 |
| Hepatitis B | 4.00E-05 | Gastric acid secretion | 2.30E-02 |
| Tyrosine metabolism | 4.40E-05 | Ascorbate and aldarate metabolism | 2.40E-02 |
| Drug metabolism - other enzymes | 4.90E-05 | Staphylococcus aureus infection | 2.40E-02 |
| Influenza A | 5.30E-05 | Salmonella infection | 2.40E-02 |
| RNA polymerase | 5.30E-05 | Peroxisome | 2.40E-02 |
| Glucagon signaling pathway | 6.30E-05 | T cell receptor signaling pathway | 2.40E-02 |
| Amphetamine addiction | 6.40E-05 | Bile secretion | 2.60E-02 |
| African trypanosomiasis | 8.30E-05 | Huntington's disease | 2.70E-02 |
| Glycolysis / Gluconeogenesis | 8.40E-05 | Platelet activation | 2.90E-02 |
| Toxoplasmosis | 8.80E-05 | Herpes simplex infection | 3.00E-02 |
| Thyroid hormone signaling pathway | 9.50E-05 | Insulin secretion | 3.10E-02 |
| Biosynthesis of antibiotics | 1.10E-04 | Small cell lung cancer | 3.10E-02 |
| Inflammatory bowel disease (IBD) | 1.10E-04 | Type I diabetes mellitus | 3.30E-02 |
| HIF-1 signaling pathway | 1.30E-04 | MAPK signaling pathway | 3.50E-02 |
| FoxO signaling pathway | 1.40E-04 | Cysteine and methionine metabolism | 3.70E-02 |
| Epstein-Barr virus infection | 1.40E-04 | Nicotinate and nicotinamide metabolism | 3.70E-02 |
| Pancreatic cancer | 1.50E-04 | NF-kappa B signaling pathway | 3.90E-02 |
| beta-Alanine metabolism | 1.60E-04 | Fatty acid metabolism | 4.10E-02 |
| Arginine and proline metabolism | 1.90E-04 | cGMP-PKG signaling pathway | 4.30E-02 |
| Prolactin signaling pathway | 2.40E-04 | Cytosolic DNA-sensing pathway | 5.00E-02 |
| Leishmaniasis | 2.40E-04 | Central carbon metabolism in cancer | 5.00E-02 |

**Table S4** The list of FDA approved drugs for SSc treatment and related targets. Totally, 42 SSc drugs and 208 different drug targets were retrieved from the DrugBank (<https://www.drugbank.ca/>).

| Drug name | Target name | Abbreviation |
| --- | --- | --- |
| Methotrexate | Dihydrofolate reductase | DHFR |
| Methotrexate | Methylenetetrahydrofolate reductase | MTHFR |
| Methotrexate | 6-phosphogluconate dehydrogenase, decarboxylating | PGD |
| Methotrexate | Folylpolyglutamate synthase, mitochondrial | PGD |
| Methotrexate | Gamma-glutamyl hydrolase | GGH |
| Methotrexate | Dihydrofolate reductase | DHFR |
| Methotrexate | Thymidylate synthase | TYMS |
| Methotrexate | Bifunctional purine biosynthesis protein PURH | ATIC |
| Methotrexate | Serum albumin | ALB |
| Methotrexate | Canalicular multispecific organic anion transporter 2 | ABCC3 |
| Methotrexate | Multidrug resistance-associated protein 4 | ABCC4 |
| Methotrexate | Multidrug resistance-associated protein 1 | ABCC1 |
| Methotrexate | Solute carrier family 22 member 6 | SLC22A6 |
| Methotrexate | Multidrug resistance-associated protein 7 | ABCC10 |
| Methotrexate | Solute carrier family 22 member 8 | SLC22A8 |
| Methotrexate | Canalicular multispecific organic anion transporter 1 | ABCC2 |
| Methotrexate | Multidrug resistance protein 1 | ABCB1 |
| Methotrexate | Solute carrier organic anion transporter family member 1A2 | SLCO1A2 |
| Methotrexate | Monocarboxylate transporter 1 | SLC16A1 |
| Methotrexate | ATP-binding cassette sub-family C member 11 | ABCC11 |
| Methotrexate | Solute carrier organic anion transporter family member 1B3 | SLCO1B3 |
| Methotrexate | Solute carrier family 22 member 11 | SLC22A11 |
| Methotrexate | Solute carrier organic anion transporter family member 1C1 | SLCO1C1 |
| Methotrexate | Solute carrier organic anion transporter family member 3A1 | SLCO3A1 |
| Methotrexate | ATP-binding cassette sub-family G member 2 | ABCG2 |
| Methotrexate | Solute carrier family 22 member 7 | SLC22A7 |
| Methotrexate | Solute carrier organic anion transporter family member 1B1 | SLCO1B1 |
| Methotrexate | Proton-coupled folate transporter | SLC46A1 |
| Methotrexate | Solute carrier organic anion transporter family member 4C1 | SLCO4C1 |
| Methotrexate | Folate transporter 1 | SLC19A1 |
| Methotrexate | Folate receptor alpha | FOLR1 |
| Methotrexate | Aldehyde oxidase | AOX1 |
| Cyclophosphamide | DNA |  |
| Cyclophosphamide | Cytochrome P450 2B6 | CYP2B6 |
| Cyclophosphamide | Cytochrome P450 2C9 | CYP2C9 |
| Cyclophosphamide | Cytochrome P450 3A4 | CYP3A4 |
| Cyclophosphamide | Cytochrome P450 2C19 | CYP2C19 |
| Cyclophosphamide | Cytochrome P450 2A6 | CYP2A6 |
| Cyclophosphamide | Cytochrome P450 2C18 | CYP2C18 |
| Cyclophosphamide | Cytochrome P450 2C8 | CYP2C8 |
| Cyclophosphamide | Cytochrome P450 2D6 | CYP2D6 |
| Cyclophosphamide | Cytochrome P450 3A7 | CYP3A7 |
| Cyclophosphamide | Cytochrome P450 3A5 | CYP3A5 |
| Cyclophosphamide | Multidrug resistance protein 1 | ABCB1 |
| Mycophenolate mofetil | Inosine-5'-monophosphate dehydrogenase 1 | IMPDH1 |
| Mycophenolate mofetil | Inosine-5'-monophosphate dehydrogenase 2 | IMPDH2 |
| Mycophenolate mofetil | UDP-glucuronosyltransferase 1-1 | UGT1A1 |
| Mycophenolate mofetil | UDP-glucuronosyltransferase 1-7 | UGT1A7 |
| Mycophenolate mofetil | UDP-glucuronosyltransferase 1-6 | UGT1A6 |
| Mycophenolate mofetil | UDP-glucuronosyltransferase 1-9 | UGT1A9 |
| Mycophenolate mofetil | UDP-glucuronosyltransferase 2B7 | UGT2B7 |
| Mycophenolate mofetil | UDP-glucuronosyltransferase 1-8 | UGT1A8 |
| Mycophenolate mofetil | UDP-glucuronosyltransferase 1-10 | UGT1A10 |
| Mycophenolate mofetil | Liver carboxylesterase 1 | CES1 |
| Mycophenolate mofetil | Cocaine esterase | CES2 |
| Mycophenolate mofetil | Cytochrome P450 3A4 | CYP3A4 |
| Mycophenolate mofetil | Cytochrome P450 3A5 | CYP3A5 |
| Mycophenolate mofetil | Cytochrome P450 2C8 | CYP2C8 |
| Mycophenolate mofetil | Serum albumin | ALB |
| Mycophenolate mofetil | Solute carrier organic anion transporter family member 1B1 | SLCO1B1 |
| Mycophenolate mofetil | Solute carrier organic anion transporter family member 1B3 | SLCO1B3 |
| Mycophenolate mofetil | Canalicular multispecific organic anion transporter 1 | ABCC2 |
| Mycophenolate mofetil | ATP-binding cassette sub-family G member 2 | ABCG2 |
| Mycophenolate mofetil | Multidrug resistance protein 1 | ABCB1 |
| Azathioprine | Hypoxanthine-guanine phosphoribosyltransferase | HPRT1 |
| Azathioprine | Thiopurine S-methyltransferase | TPMT |
| Azathioprine | Xanthine dehydrogenase/oxidase | XDH |
| Azathioprine | Glutathione S-transferase A1 | GSTA1 |
| Azathioprine | Glutathione S-transferase A2 | GSTA2 |
| Azathioprine | Glutathione S-transferase Mu 1 | GSTM1 |
| Hydroxychloroquine | DNA |  |
| Hydroxychloroquine | Toll-like receptor 7 | TLR7 |
| Hydroxychloroquine | Toll-like receptor 9 | TLR9 |
| Hydroxychloroquine | Cytochrome P450 2D6 | CYP2D6 |
| Abatacept | T-lymphocyte activation antigen CD80 | CD80 |
| Abatacept | T-lymphocyte activation antigen CD86 | CD86 |
| Rituximab | Low affinity immunoglobulin gamma Fc region receptor III-B | FCGR3B |
| Rituximab | Complement C1r subcomponent | C1R |
| Rituximab | Complement C1q subcomponent subunit A | C1QA |
| Rituximab | Complement C1q subcomponent subunit B | C1QB |
| Rituximab | Complement C1q subcomponent subunit C | C1QC |
| Rituximab | Low affinity immunoglobulin gamma Fc region receptor III-A | FCGR3A |
| Rituximab | Complement C1s subcomponent | C1S |
| Rituximab | High affinity immunoglobulin gamma Fc receptor I | FCGR1A |
| Rituximab | Low affinity immunoglobulin gamma Fc region receptor II-a | FCGR2A |
| Rituximab | Low affinity immunoglobulin gamma Fc region receptor II-b | FCGR2B |
| Rituximab | Low affinity immunoglobulin gamma Fc region receptor II-c | FCGR2C |
| Rituximab | B-lymphocyte antigen CD20 | MS4A1 |
| Belimumab | Tumor necrosis factor ligand superfamily member 13B | TNFSF13B |
| Prednisolone | Glucocorticoid receptor | NR3C1 |
| Prednisolone | Cytochrome P450 3A4 | CYP3A4 |
| Prednisolone | Cytochrome P450 2A6 | CYP2A6 |
| Prednisolone | Corticosteroid-binding globulin | SERPINA6 |
| Prednisolone | Solute carrier organic anion transporter family member 1A2 | SLCO1A2 |
| Prednisolone | Multidrug resistance protein 1 | ABCB1 |
| Prednisone | Glucocorticoid receptor | NR3C1 |
| Prednisone | Corticosteroid 11-beta-dehydrogenase isozyme 1 | HSD11B1 |
| Prednisone | Cytochrome P450 3A4 | CYP3A4 |
| Prednisone | Cytochrome P450 2C19 | CYP2C19 |
| Prednisone | Serum albumin | ALB |
| Prednisone | Multidrug resistance protein 1 | ABCB1 |
| Prednisone | Solute carrier organic anion transporter family member 1A2 | SLCO1A2 |
| Intravenous immunoglobulin | High affinity immunoglobulin gamma Fc receptor I | FCGR1A |
| Intravenous immunoglobulin | High affinity immunoglobulin gamma Fc receptor IB | FCGR1B |
| Intravenous immunoglobulin | Low affinity immunoglobulin gamma Fc region receptor II-a | FCGR2A |
| Intravenous immunoglobulin | Low affinity immunoglobulin gamma Fc region receptor II-b | FCGR2B |
| Intravenous immunoglobulin | Low affinity immunoglobulin gamma Fc region receptor II-c | FCGR2C |
| Intravenous immunoglobulin | Low affinity immunoglobulin gamma Fc region receptor III-A | FCGR3A |
| Intravenous immunoglobulin | Low affinity immunoglobulin gamma Fc region receptor III-B | FCGR3B |
| Intravenous immunoglobulin | Complement C3 | C3 |
| Intravenous immunoglobulin | Complement C4-A | C4A |
| Intravenous immunoglobulin | Complement C4-B | C4B |
| Intravenous immunoglobulin | Complement C5 | C5 |
| Bosentan | Endothelin B receptor | EDNRB |
| Bosentan | Endothelin-1 receptor | EDNRA |
| Bosentan | Cytochrome P450 3A4 | CYP3A4 |
| Bosentan | Cytochrome P450 2C9 | CYP2C9 |
| Bosentan | Bile salt export pump | ABCB11 |
| Nifedipine | Voltage-dependent L-type calcium channel subunit alpha-1C | CACNA1C |
| Nifedipine | Voltage-dependent calcium channel subunit alpha-2/delta-1 | CACNA2D1 |
| Nifedipine | Voltage-dependent L-type calcium channel subunit beta-2 | CACNB2 |
| Nifedipine | Voltage-dependent L-type calcium channel subunit alpha-1D | CACNA1D |
| Nifedipine | Voltage-dependent L-type calcium channel subunit alpha-1S | CACNA1S |
| Nifedipine | Calmodulin | CALM1 |
| Nifedipine | potassium voltage-gated channel subfamily A member 1 | KCNA1 |
| Nifedipine | Voltage-dependent T-type calcium channel subunit alpha-1H | CACNA1H |
| Nifedipine | Cytochrome P450 3A4 | CYP3A4 |
| Nifedipine | Cytochrome P450 3A5 | CYP3A5 |
| Nifedipine | Cytochrome P450 3A7 | CYP3A7 |
| Nifedipine | Cytochrome P450 1A2 | CYP1A2 |
| Nifedipine | Cytochrome P450 2A6 | CYP2A6 |
| Nifedipine | Cytochrome P450 2C8 | CYP2C8 |
| Nifedipine | Cytochrome P450 2D6 | CYP2D6 |
| Nifedipine | Cytochrome P450 2E1 | CYP2E1 |
| Nifedipine | Cytochrome P450 1A1 | CYP1A1 |
| Nifedipine | Cytochrome P450 2B6 | CYP2B6 |
| Nifedipine | Cytochrome P450 2C9 | CYP2C9 |
| Nifedipine | Canalicular multispecific organic anion transporter 2 | ABCC3 |
| Nifedipine | Multidrug resistance protein 1 | ABCB1 |
| Nifedipine | Canalicular multispecific organic anion transporter 1 | ABCC2 |
| Iloprost | Prostacyclin receptor | PTGIR |
| Iloprost | Prostaglandin E2 receptor EP1 subtype | PTGER1 |
| Iloprost | cAMP-specific 3',5'-cyclic phosphodiesterase 4A | PDE4A |
| Iloprost | cAMP-specific 3',5'-cyclic phosphodiesterase 4B | PDE4B |
| Iloprost | cAMP-specific 3',5'-cyclic phosphodiesterase 4C | PDE4C |
| Iloprost | cAMP-specific 3',5'-cyclic phosphodiesterase 4D | PDE4D |
| Iloprost | Tissue-type plasminogen activator | PLAT |
| Iloprost | Solute carrier organic anion transporter family member 2B1 | SLCO2B1 |
| Iloprost | Solute carrier organic anion transporter family member 2A1 | SLCO2A1 |
| Iloprost | Solute carrier organic anion transporter family member 3A1 | SLCO3A1 |
| Fluoxetine | Sodium-dependent serotonin transporter | SLC6A4 |
| Fluoxetine | Cytochrome P450 2C9 | CYP2C9 |
| Fluoxetine | Cytochrome P450 2D6 | CYP2D6 |
| Fluoxetine | CYP2B protein | CYP2B |
| Fluoxetine | Cytochrome P450 1A2 | CYP1A2 |
| Fluoxetine | Cytochrome P450 3A4 | CYP3A4 |
| Fluoxetine | Cytochrome P450 2C19 | CYP2C19 |
| Fluoxetine | Cytochrome P450 2B6 | CYP2B6 |
| Fluoxetine | Cytochrome P450 3A5 | CYP3A5 |
| Fluoxetine | Serum albumin | ALB |
| Fluoxetine | Alpha-1-acid glycoprotein 1 | ORM1 |
| Fluoxetine | Multidrug resistance protein 1 | ABCB1 |
| Ambrisentan | Endothelin-1 receptor | EDNRA |
| Ambrisentan | Endothelin B receptor | EDNRB |
| Ambrisentan | Cytochrome P450 3A4 | CYP3A4 |
| Ambrisentan | Cytochrome P450 2C19 | CYP2C19 |
| Ambrisentan | UDP-glucuronosyltransferase 1-9 | UGT1A9 |
| Ambrisentan | UDP-glucuronosyltransferase 2B7 | UGT2B7 |
| Ambrisentan | UDP-glucuronosyltransferase 1-3 | UGT1A3 |
| Ambrisentan | Cytochrome P450 3A5 | CYP3A5 |
| Ambrisentan | Multidrug resistance protein 1 | ABCB1 |
| Ambrisentan | Solute carrier organic anion transporter family member 1B1 | SLCO1B1 |
| Ambrisentan | Solute carrier organic anion transporter family member 1B3 | SLCO1B3 |
| Macitentan | Endothelin-1 receptor | EDNRA |
| Macitentan | Endothelin B receptor | EDNRB |
| Macitentan | Cytochrome P450 3A4 | CYP3A4 |
| Macitentan | Cytochrome P450 2C19 | CYP2C19 |
| Macitentan | Serum albumin | ALB |
| Treprostinil | Prostacyclin receptor | PTGIR |
| Treprostinil | Peroxisome proliferator-activated receptor delta | PPARD |
| Treprostinil | P2Y purinoceptor 12 | P2RY12 |
| Treprostinil | Cytochrome P450 2C9 | CYP2C9 |
| Riociguat | Guanylate cyclase soluble subunit alpha-2 | GUCY1A2 |
| Riociguat | Cytochrome P450 1A1 | CYP1A1 |
| Riociguat | Cytochrome P450 3A4 | CYP3A4 |
| Riociguat | Cytochrome P450 2C8 | CYP2C8 |
| Riociguat | Cytochrome P450 2J2 | CYP2J2 |
| Riociguat | Serum albumin | ALB |
| Riociguat | Alpha-1-acid glycoprotein 1 | ORM1 |
| Riociguat | ATP-binding cassette sub-family G member 2 | ABCG2 |
| Epoprostenol | P2Y purinoceptor 12 | P2RY12 |
| Epoprostenol | Prostacyclin receptor | PTGIR |
| Epoprostenol | Prostacyclin synthase | PTGIS |
| Epoprostenol | Cytochrome P450 2C9 | CYP2C9 |
| Vardenafil | cGMP-specific 3',5'-cyclic phosphodiesterase | PDE5A |
| Vardenafil | Retinal rod rhodopsin-sensitive cGMP 3',5'-cyclic phosphodiesterase subunit gamma | PDE6G |
| Vardenafil | Retinal cone rhodopsin-sensitive cGMP 3',5'-cyclic phosphodiesterase subunit gamma | PDE6H |
| Vardenafil | Cytochrome P450 3A4 | CYP3A4 |
| Vardenafil | Cytochrome P450 3A5 | CYP3A5 |
| Tadalafil | Dual 3',5'-cyclic-AMP and -GMP phosphodiesterase 11A | PDE11A |
| Tadalafil | Cytochrome P450 3A4 | CYP3A4 |
| Tadalafil | cGMP-specific 3',5'-cyclic phosphodiesterase | PDE5A |
| Sildenafil | cGMP-specific 3',5'-cyclic phosphodiesterase | PDE5A |
| Sildenafil | Retinal rod rhodopsin-sensitive cGMP 3',5'-cyclic phosphodiesterase subunit gamma | PDE6G |
| Sildenafil | Retinal cone rhodopsin-sensitive cGMP 3',5'-cyclic phosphodiesterase subunit gamma | PDE6H |
| Sildenafil | Cytochrome P450 3A4 | CYP3A4 |
| Sildenafil | Cytochrome P450 3A5 | CYP3A5 |
| Sildenafil | Cytochrome P450 3A7 | CYP3A7 |
| Sildenafil | Cytochrome P450 2C9 | CYP2C9 |
| Sildenafil | Cytochrome P450 2C19 | CYP2C19 |
| Sildenafil | Cytochrome P450 2D6 | CYP2D6 |
| Sildenafil | Cytochrome P450 1A1 | CYP1A1 |
| Sildenafil | Cytochrome P450 2E1 | CYP2E1 |
| Sildenafil | Multidrug resistance-associated protein 4 | ABCC4 |
| Sildenafil | Multidrug resistance-associated protein 5 | ABCC5 |
| Sildenafil | Multidrug resistance-associated protein 7 | ABCC10 |
| Imatinib | BCR/ABL fusion protein isoform X9 | BCR/ABL fusion |
| Imatinib | Mast/stem cell growth factor receptor Kit | KIT |
| Imatinib | RET proto-oncogene | RET |
| Imatinib | High affinity nerve growth factor receptor | NTRK1 |
| Imatinib | Macrophage colony-stimulating factor 1 receptor | CSF1R |
| Imatinib | Platelet-derived growth factor receptor alpha | PDGFRA |
| Imatinib | Epithelial discoidin domain-containing receptor 1 | DDR1 |
| Imatinib | Tyrosine-protein kinase ABL1 | ABL1 |
| Imatinib | Platelet-derived growth factor receptor beta | PDGFRB |
| Imatinib | Cytochrome P450 3A4 | CYP3A4 |
| Imatinib | Cytochrome P450 3A5 | CYP3A5 |
| Imatinib | Cytochrome P450 3A7 | CYP3A7 |
| Imatinib | Cytochrome P450 1A2 | CYP1A2 |
| Imatinib | Cytochrome P450 2C9 | CYP2C9 |
| Imatinib | Cytochrome P450 2D6 | CYP2D6 |
| Imatinib | Cytochrome P450 2C19 | CYP2C19 |
| Imatinib | Prostaglandin G/H synthase 1 | PTGS1 |
| Imatinib | Serum albumin | ALB |
| Imatinib | Alpha-1-acid glycoprotein 1 | ORM1 |
| Imatinib | Solute carrier family 22 member 1 | SLC22A1 |
| Imatinib | Multidrug resistance protein 1 | ABCB1 |
| Imatinib | Solute carrier family 22 member 2 | SLC22A2 |
| Imatinib | ATP-binding cassette sub-family G member 2 | ABCG2 |
| Imatinib | ATP-binding cassette sub-family A member 3 | ABCA3 |
| Imatinib | Tyrosine-protein kinase ABL1 | ABL1 |
| Nilotinib | Mast/stem cell growth factor receptor Kit | KIT |
| Nilotinib | Cytochrome P450 3A4 | CYP3A4 |
| Nilotinib | Cytochrome P450 2C8 | CYP2C8 |
| Nilotinib | Cytochrome P450 2C9 | CYP2C9 |
| Nilotinib | Cytochrome P450 2D6 | CYP2D6 |
| Nilotinib | Cytochrome P450 2B6 | CYP2B6 |
| Nilotinib | Multidrug resistance protein 1 | ABCB1 |
| Nilotinib | ATP-binding cassette sub-family G member 2 | ABCG2 |
| Nilotinib | UDP-glucuronosyltransferase 1-1 | UGT1A1 |
| Nintedanib | Vascular endothelial growth factor receptor 1 | FLT1 |
| Nintedanib | Vascular endothelial growth factor receptor 2 | KDR |
| Nintedanib | Vascular endothelial growth factor receptor 3 | FLT4 |
| Nintedanib | Platelet derived growth factor receptor alpha |  |
| Nintedanib | Platelet derived growth factor receptor beta |  |
| Nintedanib | Fibroblast growth factor receptor 1 | FGFR1 |
| Nintedanib | Fibroblast growth factor receptor 2 | FGFR2 |
| Nintedanib | Fibroblast growth factor receptor 3 | FGFR3 |
| Nintedanib | Receptor-type tyrosine-protein kinase FLT3 | FLT3 |
| Nintedanib | Tyrosine-protein kinase Lck | LCK |
| Nintedanib | Tyrosine-protein kinase Lyn | LYN |
| Nintedanib | Proto-oncogene tyrosine-protein kinase Src | SRC |
| Nintedanib | Multidrug resistance protein 1 | ABCB1 |
| Dasatinib | Tyrosine-protein kinase ABL1 | ABL1 |
| Dasatinib | Proto-oncogene tyrosine-protein kinase Src | SRC |
| Dasatinib | Ephrin type-A receptor 2 | EPHA2 |
| Dasatinib | Tyrosine-protein kinase Lck | LCK |
| Dasatinib | Tyrosine-protein kinase Yes | YES1 |
| Dasatinib | Mast/stem cell growth factor receptor Kit | KIT |
| Dasatinib | Platelet-derived growth factor receptor beta | PDGFRB |
| Dasatinib | Signal transducer and activator of transcription 5B | STAT5B |
| Dasatinib | Abelson tyrosine-protein kinase 2 | ABL2 |
| Dasatinib | Tyrosine-protein kinase Fyn | FYN |
| Dasatinib | Cytochrome P450 3A4 | CYP3A4 |
| Dasatinib | Cytochrome P450 1A1 | CYP1A1 |
| Dasatinib | Cytochrome P450 1A2 | CYP1A2 |
| Dasatinib | Cytochrome P450 1B1 | CYP1B1 |
| Dasatinib | Cytochrome P450 3A5 | CYP3A5 |
| Dasatinib | Dimethylaniline monooxygenase [N-oxide-forming] 3 | FMO3 |
| Dasatinib | Multidrug resistance protein 1 | ABCB1 |
| Dasatinib | ATP-binding cassette sub-family G member 2 | ABCG2 |
| Tocilizumab | Interleukin-6 receptor subunit alpha | IL6R |
| Botulinum toxin AA | Synaptosomal-associated protein 25 | SNAP25 |
| Botulinum toxin AA | Rho-related GTP-binding protein RhoB | RHOB |
| Rosuvastin | 3-hydroxy-3-methylglutaryl-coenzyme A reductase | HMGCR |
| Rosuvastin | Cytochrome P450 2C9 | CYP2C9 |
| Rosuvastin | Cytochrome P450 2C19 | CYP2C19 |
| Rosuvastin | Cytochrome P450 3A4 | CYP3A4 |
| Rosuvastin | Cytochrome P450 3A5 | CYP3A5 |
| Rosuvastin | Serum albumin | ALB |
| Rosuvastin | Multidrug resistance-associated protein 1 | ABCC1 |
| Rosuvastin | Multidrug resistance-associated protein 4 | ABCC4 |
| Rosuvastin | Solute carrier organic anion transporter family member 1A2 | SLCO1A2 |
| Rosuvastin | Solute carrier organic anion transporter family member 1B1 | SLCO1B1 |
| Rosuvastin | Solute carrier organic anion transporter family member 1B3 | SLCO1B3 |
| Rosuvastin | Solute carrier organic anion transporter family member 2B1 | SLCO2B1 |
| Rosuvastin | Cystine/glutamate transporter | SLC7A11 |
| Rosuvastin | Bile salt export pump | ABCB11 |
| Rosuvastin | ATP-binding cassette sub-family G member 2 | ABCG2 |
| Dabigatran etexilate | Liver carboxylesterase 1 | CES1 |
| Dabigatran etexilate | Cocaine esterase | CES2 |
| Dabigatran etexilate | UDP-glucuronosyltransferase 1-9 | UGT1A9 |
| Dabigatran etexilate | UDP-glucuronosyltransferase 2B7 | UGT2B7 |
| Dabigatran etexilate | UDP-glucuronosyltransferase 2B15 | UGT2B15 |
| Dabigatran etexilate | Ribosyldihydronicotinamide dehydrogenase [quinone] | NQO2 |
| Dabigatran etexilate | Multidrug resistance protein 1 | ABCB1 |
| Dabigatran etexilate | Prothrombin | F2 |
| Rosiglitazone | Peroxisome proliferator-activated receptor gamma | PPARG |
| Rosiglitazone | Long-chain-fatty-acid--CoA ligase 4 | ACSL4 |
| Rosiglitazone | Cytochrome P450 2C8 | CYP2C8 |
| Rosiglitazone | Cytochrome P450 2C9 | CYP2C9 |
| Rosiglitazone | Prostaglandin G/H synthase 1 | PTGS1 |
| Rosiglitazone | Cytochrome P450 1A2 | CYP1A2 |
| Rosiglitazone | Cytochrome P450 2A6 | CYP2A6 |
| Rosiglitazone | Cytochrome P450 2C19 | CYP2C19 |
| Rosiglitazone | Cytochrome P450 2D6 | CYP2D6 |
| Rosiglitazone | Serum albumin | ALB |
| Rosiglitazone | Solute carrier organic anion transporter family member 1B1 | SLCO1B1 |
| Pioglitazone | Peroxisome proliferator-activated receptor gamma | PPARG |
| Pioglitazone | Cytochrome P450 2C8 | CYP2C8 |
| Pioglitazone | Cytochrome P450 3A4 | CYP3A4 |
| Pioglitazone | Prostaglandin G/H synthase 1 | PTGS1 |
| Pioglitazone | Cytochrome P450 2C19 | CYP2C19 |
| Pioglitazone | Cytochrome P450 2C9 | CYP2C9 |
| Pioglitazone | Cytochrome P450 2D6 | CYP2D6 |
| Pioglitazone | Solute carrier organic anion transporter family member 1B3 | SLCO1B3 |
| Pioglitazone | Solute carrier organic anion transporter family member 1B1 | SLCO1B1 |
| Captopril | Angiotensin-converting enzyme | ACE |
| Captopril | 72 kDa type IV collagenase | MMP2 |
| Captopril | Matrix metalloproteinase-9 | MMP9 |
| Captopril | Leukotriene A-4 hydrolase | LTA4H |
| Captopril | Cytochrome P450 2D6 | CYP2D6 |
| Captopril | Serum albumin | ALB |
| Captopril | Multidrug resistance protein 1 | ABCB1 |
| Captopril | Solute carrier family 15 member 1 | SLC15A1 |
| Captopril | Solute carrier family 22 member 6 | SLC22A6 |
| Cisapride | 5-hydroxytryptamine receptor 4 | HTR4 |
| Cisapride | 5-hydroxytryptamine receptor 3A | HTR3A |
| Cisapride | 5-hydroxytryptamine receptor 2A | HTR2A |
| Cisapride | Potassium voltage-gated channel subfamily H member 2 | KCNH2 |
| Cisapride | Cytochrome P450 3A4 | CYP3A4 |
| Cisapride | Cytochrome P450 3A5 | CYP3A5 |
| Cisapride | Cytochrome P450 3A7 | CYP3A7 |
| Cisapride | Cytochrome P450 1A2 | CYP1A2 |
| Cisapride | Cytochrome P450 2A6 | CYP2A6 |
| Cisapride | Cytochrome P450 2B6 | CYP2B6 |
| Cisapride | Cytochrome P450 2C19 | CYP2C19 |
| Cisapride | Cytochrome P450 2C8 | CYP2C8 |
| Cisapride | Cytochrome P450 2C9 | CYP2C9 |
| Cisapride | Cytochrome P450 2D6 | CYP2D6 |
| Amoxicillin-clavulanic acid | Penicillin-binding protein 1A | pbpA |
| Amoxicillin-clavulanic acid | Cytochrome P450 2C19 | CYP2C19 |
| Amoxicillin-clavulanic acid | Solute carrier family 15 member 1 | SLC15A1 |
| Amoxicillin-clavulanic acid | Solute carrier family 15 member 2 | SLC15A2 |
| Amoxicillin-clavulanic acid | Solute carrier family 22 member 6 | SLC22A6 |
| Amoxicillin-clavulanic acid | Beta-lactamase | blaZ |
| Omeprazole | Potassium-transporting ATPase alpha chain 1 | ATP4A |
| Omeprazole | Cytochrome P450 2C19 | CYP2C19 |
| Omeprazole | Cytochrome P450 3A4 | CYP3A4 |
| Omeprazole | Cytochrome P450 1A2 | CYP1A2 |
| Omeprazole | Cytochrome P450 2C9 | CYP2C9 |
| Omeprazole | Cytochrome P450 2C18 | CYP2C18 |
| Omeprazole | Cytochrome P450 2C8 | CYP2C8 |
| Omeprazole | Cytochrome P450 2D6 | CYP2D6 |
| Omeprazole | Cholesterol side-chain cleavage enzyme, mitochondrial | CYP11A1 |
| Omeprazole | Cytochrome P450 1A1 | CYP1A1 |
| Omeprazole | Cytochrome P450 1B1 | CYP1B1 |
| Omeprazole | Canalicular multispecific organic anion transporter 2 | ABCC3 |
| Omeprazole | Multidrug resistance protein 1 | ABCB1 |
| Omeprazole | ATP-binding cassette sub-family G member 2 | ABCG2 |
| Ciprofloxacin | DNA topoisomerase 4 subunit A | parC |
| Ciprofloxacin | DNA gyrase subunit A | gyrA |
| Ciprofloxacin | DNA topoisomerase 2-alpha | TOP2A |
| Ciprofloxacin | Potassium voltage-gated channel subfamily H member 2 | KCNH2 |
| Ciprofloxacin | DNA gyrase subunit A | gyrA |
| Ciprofloxacin | DNA topoisomerase 4 subunit A | parC |
| Ciprofloxacin | DNA topoisomerase 4 subunit B | parE |
| Ciprofloxacin | DNA gyrase subunit A | gyrA |
| Ciprofloxacin | Multidrug resistance protein MdtK | mdtK |
| Ciprofloxacin | Gyrase A |  |
| Ciprofloxacin | DNA gyrase subunit A | gyrA |
| Ciprofloxacin | Cytochrome P450 1A2 | CYP1A2 |
| Ciprofloxacin | Cytochrome P450 3A4 | CYP3A4 |
| Ciprofloxacin | Cytochrome P450 3A5 | CYP3A5 |
| Ciprofloxacin | Cytochrome P450 3A7 | CYP3A7 |
| Ciprofloxacin | Multidrug resistance protein 1 | ABCB1 |
| Loperamide | Mu-type opioid receptor | OPRM1 |
| Loperamide | Delta-type opioid receptor | OPRD1 |
| Loperamide | Kappa-type opioid receptor | OPRK1 |
| Loperamide | Voltage-dependent P/Q-type calcium channel subunit alpha-1A | CACNA1A |
| Loperamide | Pro-opiomelanocortin | POMC |
| Loperamide | Calmodulin | CALM1 |
| Loperamide | Cytochrome P450 3A4 | CYP3A4 |
| Loperamide | Cytochrome P450 2C8 | CYP2C8 |
| Loperamide | Cytochrome P450 2B6 | CYP2B6 |
| Loperamide | Cytochrome P450 2D6 | CYP2D6 |
| Loperamide | Multidrug resistance protein 1 | ABCB1 |
| Carvedilol | Beta-1 adrenergic receptor | ADRB1 |
| Carvedilol | Alpha-1A adrenergic receptor | ADRA1A |
| Carvedilol | NADH dehydrogenase [ubiquinone] 1 subunit C2 | NDUFC2 |
| Carvedilol | Beta-2 adrenergic receptor | ADRB2 |
| Carvedilol | Vascular endothelial growth factor A | VEGFA |
| Carvedilol | Natriuretic peptides B | NPPB |
| Carvedilol | Gap junction alpha-1 protein | GJA1 |
| Carvedilol | Potassium voltage-gated channel subfamily H member 2 | KCNH2 |
| Carvedilol | Vascular cell adhesion protein 1 | VCAM1 |
| Carvedilol | Alpha-1D adrenergic receptor | ADRA1D |
| Carvedilol | Alpha-1B adrenergic receptor | ADRA1B |
| Carvedilol | Alpha-2C adrenergic receptor | ADRA2C |
| Carvedilol | Alpha-2B adrenergic receptor | ADRA2B |
| Carvedilol | Alpha-2A adrenergic receptor | ADRA2A |
| Carvedilol | E-selectin | SELE |
| Carvedilol | Hypoxia-inducible factor 1-alpha | HIF1A |
| Carvedilol | Cytochrome P450 2C9 | CYP2C9 |
| Carvedilol | Cytochrome P450 2D6 | CYP2D6 |
| Carvedilol | Xanthine dehydrogenase/oxidase | XDH |
| Carvedilol | Cytochrome P450 1A2 | CYP1A2 |
| Carvedilol | Cytochrome P450 3A4 | CYP3A4 |
| Carvedilol | Cytochrome P450 1A1 | CYP1A1 |
| Carvedilol | Cytochrome P450 2E1 | CYP2E1 |
| Carvedilol | Prostaglandin G/H synthase 1 | PTGS1 |
| Carvedilol | Multidrug resistance protein 1 | ABCB1 |
| Spironolactone | Mineralocorticoid receptor | NR3C2 |
| Spironolactone | Androgen receptor | AR |
| Spironolactone | Progesterone receptor | PGR |
| Spironolactone | Glucocorticoid receptor | NR3C1 |
| Spironolactone | Cytochrome P450 11B2, mitochondrial | CYP11B2 |
| Spironolactone | 17alpha-hydroxylase |  |
| Spironolactone | 17,20-desmolase |  |
| Spironolactone | 3-oxo-5-alpha-steroid 4-dehydrogenase (Protein  Group) |  |
| Spironolactone | Sex hormone-binding globulin | SHBG |
| Spironolactone | Voltage-dependent calcium channel gamma-1 subunit | CACNG1 |
| Spironolactone | Dihydrotestosterone receptor |  |
| Spironolactone | Cytochrome P450 2C8 | CYP2C8 |
| Spironolactone | Cytochrome P450 11B1, mitochondrial | CYP11B1 |
| Spironolactone | Canalicular multispecific organic anion transporter 1 | ABCC2 |
| Spironolactone | Multidrug resistance protein 1 | ABCB1 |
| Spironolactone | Solute carrier organic anion transporter family member 1A2 | SLCO1A2 |
| Furosemide | Solute carrier family 12 member 1 | SLC12A1 |
| Furosemide | Carbonic anhydrase 2 | CA2 |
| Furosemide | 6-phosphogluconate dehydrogenase, decarboxylating | PGD |
| Furosemide | Serum albumin | ALB |
| Furosemide | Solute carrier family 22 member 6 | SLC22A6 |
| Furosemide | Solute carrier family 22 member 5 | SLC22A5 |
| Furosemide | Solute carrier family 22 member 8 | SLC22A8 |
| Furosemide | Canalicular multispecific organic anion transporter 1 | ABCC2 |
| Furosemide | Solute carrier organic anion transporter family member 2A1 | SLCO2A1 |
| Furosemide | Solute carrier family 22 member 11 | SLC22A11 |

**Table S5** The list of reported SSc disease genes. 131 SSc gene proteins were collected from 3 databases: OMIM (<http://www.omim.org>), GAD (<http://geneticassociationdb.nih.gov/>), and CATALOG (http://www.ebi.ac.uk/gwas/).

| Abbreviation | Database | Abbreviation | Database | Abbreviation | Database |
| --- | --- | --- | --- | --- | --- |
| HLA-DRA | Catalog | C2 | GAD | IL10RB | GAD |
| NOTCH4 | Catalog | ACE | GAD | IFNG | GAD |
| HLA-DPA1 | Catalog | TNF | GAD | HTR2A | GAD |
| TNPO | Catalog | TAP2 | GAD | HLA-DPB2 | GAD |
| IRF8 | Catalog | TAP1 | GAD | HIF1A | GAD |
| ITGAM | Catalog | SPARC | GAD | FLT1 | GAD |
| PSORS1C1 | Catalog | PTPRC | GAD | ESR2 | GAD |
| TNFAIP3 | Catalog | PDGFB | GAD | ESR1 | GAD |
| CD247 | Catalog | NAT2 | GAD | IL23R | GAD |
| BLK | Catalog | MMP1 | GAD | BANK1 | GAD |
| TNIP1 | Catalog | IL1A | GAD | TRBV3-1 | GAD |
| CSK | Catalog | HLA-DRB4 | GAD | TRBV20-1 | GAD |
| ESYT1 | Catalog | HLA | GAD | FAS | GAD |
| UHRF1BP1 | Catalog | TNFRSF1B | GAD | TBX21 | GAD |
| GRB10 | Catalog | C4 | GAD | CXCL12 | GAD |
| RHOB | Catalog | CYP2D6 | GAD | CD19 | GAD,OMIM |
| SOX5 | Catalog | HLA-DRB5 | GAD | FBN1 | GAD,OMIM |
| NMNAT2 | Catalog | NOS3 | GAD | CCL2 | GAD,OMIM |
| IKZF3 | Catalog | TERC | GAD | CTGF | GAD,OMIM |
| PXK | Catalog | TGFB | GAD | TGFB1 | GAD,OMIM |
| KIAA0319L | Catalog | AIF1 | GAD | SPAG9 | OMIM |
| JAZF1 | Catalog | AIRE | GAD | PF4 | OMIM |
| RPL41 | Catalog | CCL5 | GAD | CCL7 | OMIM |
| DNPEP | Catalog | CCR7 | GAD | FBL | OMIM |
| SAMD9L | Catalog | CD22 | GAD | SDC2 | OMIM |
| ATG5 | Catalog | CX3CR1 | GAD | CD28 | OMIM |
| EHF | Catalog | ENG | GAD | LBR | OMIM |
| IRF7 | Catalog | IL10 | GAD | RPP30 | OMIM |
| HLA-DQB1 | Catalog, GAD | IL13 | GAD | RPP38 | OMIM |
| HLA-DPB1 | Catalog, GAD | IL1B | GAD | RPL7 | OMIM |
| IRF5 | Catalog, GAD | IL1RN | GAD | GDF6 | OMIM |
| HLA-DQA1 | Catalog, GAD | IL2 | GAD | PDGFRB | OMIM |
| TNPO3 | Catalog, GAD | IL23A | GAD | EXOSC9 | OMIM |
| TNFSF4 | Catalog, GAD | LTA | GAD | EXOSC10 | OMIM |
| HLA-DRB1 | Catalog, GAD, OMIM | NOS2A | GAD | SSSCA1 | OMIM |
| STAT4 | Catalog, GAD, OMIM | PTPN22 | GAD | TEX101 | OMIM |
| FN1 | GAD | VEGFA | GAD | SMAD7 | OMIM |
| CYP2E1 | GAD | CD86 | GAD | SMAD3 | OMIM |
| CYP2C9 | GAD | IL13RA2 | GAD | MIRLET7A1 | OMIM |
| CYP2C19 | GAD | IL6 | GAD | POLR3A | OMIM |
| CYP1A1 | GAD | IL8 | GAD | FXR1 | OMIM |
| CYBA | GAD | MTHFR | GAD | CENPC | OMIM |
| CTLA4 | GAD | KDR | GAD | PYGM | OMIM |
| COL1A2 | GAD | IL1R1 | GAD |  |  |

**Table S6** The list of the shared targets of formula targets, disease genes, and drug targets. 10 formula targets were confirmed to overlap with disease genes and drug targets.

| Target name | Abbreviation |
| --- | --- |
| Methylenetetrahydrofolate reductase | MTHFR |
| Angiotensin-converting enzyme | ACE |
| 5-hydroxytryptamine receptor 2A | HTR2A |
| Cytochrome P450 2C19 | CYP2C19 |
| Vascular endothelial growth factor A | VEGFA |
| Hypoxia-inducible factor 1-alpha | HIF1A |
| Cytochrome P450 2C9 | CYP2C9 |
| Cytochrome P450 2D6 | CYP2D6 |
| Cytochrome P450 1A1 | CYP1A1 |
| Cytochrome P450 2E1 | CYP2E1 |

**Table S7** GO enrichment analysis of PPI modules. Each module was performed the GO enrichment analysis by online website DAVID Bioinformatics Resources 6.8 (<https://david.ncifcrf.gov/>), 22 modules and 126 GO terms were filtered according to P-value ≤ 0.05.

| Module ranking | GO Terms | Genes | P-value |
| --- | --- | --- | --- |
| Module 1 | fibroblast growth factor receptor signaling pathway | 11 | 3.40E-20 |
|  | positive regulation of epithelial cell migration | 2 | 3.10E-02 |
| Module 2 | SMAD protein signal transduction | 3 | 3.00E-03 |
|  | transforming growth factor beta receptor signaling pathway | 3 | 6.40E-03 |
|  | SMAD protein complex | 2 | 9.60E-03 |
|  | SMAD protein complex assembly | 2 | 1.00E-02 |
|  | regulation of transforming growth factor beta receptor signaling pathway | 2 | 2.60E-02 |
|  | positive regulation of transforming growth factor beta receptor signaling pathway | 2 | 3.10E-02 |
|  | positive regulation of epithelial to mesenchymal transition | 2 | 4.20E-02 |
|  | positive regulation of smooth muscle cell proliferation | 2 | 7.60E-02 |
| Module 3 | extracellular matrix | 17 | 7.20E-08 |
|  | positive regulation of nitric oxide biosynthetic process | 5 | 1.30E-03 |
|  | cellular response to interleukin-4 | 4 | 2.30E-03 |
|  | regulation of inflammatory response | 5 | 5.20E-03 |
|  | receptor signaling protein tyrosine kinase activity | 3 | 5.20E-03 |
|  | regulation of interferon-gamma-mediated signaling pathway | 3 | 8.80E-03 |
|  | regulation of cytokine production | 3 | 8.80E-03 |
|  | MHC class II protein complex binding | 3 | 1.30E-02 |
|  | platelet formation | 3 | 1.80E-02 |
|  | positive regulation of type I interferon production | 4 | 1.90E-02 |
|  | PI3K-Akt signaling pathway | 14 | 2.10E-02 |
|  | positive regulation of fibroblast proliferation | 4 | 2.20E-02 |
|  | transmembrane receptor protein tyrosine kinase signaling pathway | 5 | 2.20E-02 |
|  | positive regulation of smooth muscle cell proliferation | 4 | 2.90E-02 |
|  | platelet-derived growth factor receptor signaling pathway | 3 | 4.10E-02 |
|  | negative regulation of smooth muscle cell proliferation | 3 | 4.10E-02 |
|  | positive regulation of reactive oxygen species metabolic process | 3 | 4.30E-02 |
|  | vascular endothelial growth factor receptor signaling pathway | 4 | 4.60E-02 |
| Module4 | steroid hormone receptor activity | 7 | 2.70E-05 |
|  | steroid hormone mediated signaling pathway | 7 | 3.30E-05 |
|  | positive regulation of NF-kappaB transcription factor activity | 7 | 3.20E-03 |
|  | positive regulation of B cell proliferation | 3 | 6.60E-02 |
| Module5 | positive regulation of type I interferon production | 8 | 3.70E-10 |
|  | positive regulation of interferon-beta production | 4 | 1.00E-04 |
|  | positive regulation of innate immune response | 3 | 1.70E-03 |
|  | innate immune response | 7 | 3.30E-03 |
|  | positive regulation of fibroblast proliferation | 3 | 1.40E-02 |
|  | epidermal growth factor receptor signaling pathway | 3 | 1.60E-02 |
| Module6 | extracellular matrix | 7 | 4.20E-04 |
| Module7 | T cell receptor signaling pathway | 12 | 5.60E-09 |
|  | Fc-epsilon receptor signaling pathway | 10 | 3.70E-06 |
|  | negative regulation of epidermal growth factor receptor signaling pathway | 5 | 1.00E-04 |
|  | response to oxidative stress | 7 | 1.10E-04 |
|  | positive regulation of epidermal growth factor receptor signaling pathway | 4 | 5.70E-04 |
|  | activation of JUN kinase activity | 4 | 2.20E-03 |
|  | B cell receptor signaling pathway | 4 | 6.00E-03 |
|  | activation of MAPK activity | 5 | 6.30E-03 |
|  | positive regulation of canonical Wnt signaling pathway | 5 | 9.30E-03 |
|  | innate immune response | 8 | 2.80E-02 |
|  | positive regulation of nitric oxide biosynthetic process | 3 | 3.50E-02 |
|  | platelet activation | 4 | 4.40E-02 |
| Module8 | Fc-epsilon receptor signaling pathway | 3 | 1.50E-02 |
|  | positive regulation of tyrosine phosphorylation of Stat3 protein | 2 | 4.00E-02 |
| Module9 | regulation of tumor necrosis factor-mediated signaling pathway | 4 | 1.50E-04 |
|  | positive regulation of NF-kappaB transcription factor activity | 5 | 1.10E-03 |
|  | I-kappaB kinase/NF-kappaB signaling | 4 | 1.20E-03 |
|  | T cell receptor signaling pathway | 5 | 1.70E-03 |
|  | inflammatory response | 7 | 1.90E-03 |
|  | positive regulation of I-kappaB kinase/NF-kappaB signaling | 5 | 2.30E-03 |
|  | innate immune response | 6 | 1.60E-02 |
|  | Fc-epsilon receptor signaling pathway | 4 | 2.40E-02 |
|  | CCR5 chemokine receptor binding | 2 | 2.70E-02 |
|  | positive regulation of interleukin-2 production | 2 | 4.70E-02 |
| Module10 | T cell receptor signaling pathway | 10 | 2.30E-02 |
| Module11 | T cell receptor signaling pathway | 10 | 8.10E-06 |
|  | positive regulation of interferon-gamma production | 5 | 7.80E-04 |
|  | positive regulation of memory T cell differentiation | 3 | 1.20E-03 |
|  | positive regulation of T-helper 17 type immune response | 3 | 1.20E-03 |
|  | positive regulation of activated T cell proliferation | 4 | 1.80E-03 |
|  | positive regulation of T-helper 1 type immune response | 3 | 2.20E-03 |
|  | antigen processing and presentation of exogenous peptide antigen via MHC class II | 5 | 9.70E-03 |
|  | Wnt signaling pathway, planar cell polarity pathway | 5 | 9.70E-03 |
|  | positive regulation of T cell activation | 3 | 1.10E-02 |
|  | interleukin-23 receptor complex | 2 | 1.70E-02 |
|  | positive regulation of interleukin-12 production | 3 | 2.10E-02 |
|  | inflammatory response | 9 | 2.20E-02 |
|  | tumor necrosis factor-mediated signaling pathway | 5 | 2.20E-02 |
|  | Fc-epsilon receptor signaling pathway | 6 | 2.30E-02 |
|  | toll-like receptor signaling pathway | 3 | 2.50E-02 |
|  | interleukin-2 receptor activity | 2 | 2.70E-02 |
|  | interleukin-2 binding | 2 | 2.70E-02 |
|  | vascular endothelial growth factor receptor signaling pathway | 4 | 2.80E-02 |
|  | positive regulation of protein serine/threonine kinase activity | 3 | 4.00E-02 |
| Module12 | interferon-gamma-mediated signaling pathway | 4 | 3.00E-04 |
|  | antigen processing and presentation of peptide or polysaccharide antigen via MHC class II | 3 | 4.40E-04 |
|  | MHC class II protein complex | 3 | 6.30E-04 |
|  | regulation of interleukin-10 secretion | 2 | 5.50E-03 |
|  | regulation of interleukin-4 production | 2 | 7.40E-03 |
|  | immunoglobulin production involved in immunoglobulin mediated immune response | 2 | 9.20E-03 |
|  | peptide antigen assembly with MHC class II protein complex | 2 | 9.20E-03 |
|  | antigen processing and presentation of exogenous peptide antigen via MHC class II | 3 | 1.20E-02 |
|  | transforming growth factor beta receptor signaling pathway | 3 | 1.20E-02 |
|  | regulation of fibroblast migration | 2 | 1.80E-02 |
|  | T-helper 1 type immune response | 2 | 2.20E-02 |
|  | MHC class II receptor activity | 2 | 2.70E-02 |
|  | inflammatory response to antigenic stimulus | 2 | 2.70E-02 |
|  | T cell receptor signaling pathway | 4 | 3.00E-02 |
|  | immune response | 2 | 4.20E-02 |
|  | negative regulation of interferon-gamma production | 2 | 5.00E-02 |
| Module13 | protein serine/threonine phosphatase activity | 2 | 3.40E-02 |
| Module14 | cellular response to glucagon stimulus | 2 | 9.50E-03 |
| Module16 | Wnt signaling pathway, calcium modulating pathway | 2 | 7.10E-04 |
|  | T cell activation | 2 | 9.30E-03 |
|  | Fc-epsilon receptor signaling pathway | 2 | 1.10E-02 |
| Module17 | cytosol | 3 | 3.30E-02 |
| Module19 | [metalloendopeptidase activity](http://www.ebi.ac.uk/QuickGO/GTerm?id=GO:0004222) | 2 | 3.30E-02 |
|  | [poly(A) RNA binding](http://www.ebi.ac.uk/QuickGO/GTerm?id=GO:0044822) | 2 | 3.90E-02 |
| Module20 | membrane | 6 | 1.00E-03 |
|  | extracellular exosome | 6 | 3.20E-03 |
|  | cell differentiation | 3 | 1.90E-02 |
| Module21 | nucleus | 4 | 2.60E-02 |
| Module22 | nucleoplasm | 9 | 1.80E-04 |
|  | cytosol | 9 | 6.40E-04 |
|  | cytoplasm | 10 | 2.80E-03 |
|  | mesenchyme migration | 2 | 3.90E-03 |
| Module 23 | platelet-derived growth factor receptor binding | 2 | 8.00E-03 |
|  | platelet-derived growth factor receptor signaling pathway | 2 | 1.50E-02 |
|  | ribosome biogenesis | 2 | 1.90E-02 |
|  | positive regulation of cell proliferation | 3 | 2.40E-02 |
|  | protein binding | 9 | 2.60E-02 |
| Module 24 | U6 snRNP | 3 | 1.30E-06 |
|  | spliceosomal complex | 3 | 2.60E-04 |
|  | RNA splicing | 3 | 9.50E-04 |
|  | mRNA splicing, via spliceosome | 3 | 1.70E-03 |
|  | nucleoplasm | 5 | 2.40E-03 |

**Table S8**  Source of 121 microarrays. 121 microarrays were downloaded from GEO database, including 15 normal samples, 61 SSc disease samples and 45 SSc treatment samples.

| **GSM ID** | **Group** | **GSE ID** | **GSM ID** | **Group** | **GSE ID** |
| --- | --- | --- | --- | --- | --- |
| GSM1944594 | SSc_ONLY | GSE75173 | GSM1944595 | SSc_TREAT | GSE75173 |
| GSM1944596 | SSc_ONLY | GSE75173 | GSM1944597 | SSc_TREAT | GSE75173 |
| GSM1944598 | SSc_ONLY | GSE75173 | GSM1944599 | SSc_TREAT | GSE75173 |
| GSM1944600 | SSc_ONLY | GSE75173 | GSM1944601 | SSc_TREAT | GSE75173 |
| GSM1944602 | SSc_ONLY | GSE75173 | GSM1944603 | SSc_TREAT | GSE75173 |
| GSM1944604 | SSc_ONLY | GSE75173 | GSM1944605 | SSc_TREAT | GSE75173 |
| GSM1944606 | SSc_ONLY | GSE75173 | GSM1944607 | SSc_TREAT | GSE75173 |
| GSM1944608 | SSc_ONLY | GSE75173 | GSM1944609 | SSc_TREAT | GSE75173 |
| GSM1944610 | SSc_ONLY | GSE75173 | GSM1944611 | SSc_TREAT | GSE75173 |
| GSM1944612 | SSc_ONLY | GSE75173 | GSM1944613 | SSc_TREAT | GSE75173 |
| GSM1003068 | SSc_ONLY | GSE40839 | GSM1328198 | SSc_TREAT | GSE55036 |
| GSM1003069 | SSc_ONLY | GSE40839 | GSM1328199 | SSc_TREAT | GSE55036 |
| GSM1003070 | SSc_ONLY | GSE40839 | GSM1328200 | SSc_TREAT | GSE55036 |
| GSM1003071 | SSc_ONLY | GSE40839 | GSM1328202 | SSc_TREAT | GSE55036 |
| GSM1003072 | SSc_ONLY | GSE40839 | GSM1328203 | SSc_TREAT | GSE55036 |
| GSM1003073 | SSc_ONLY | GSE40839 | GSM1328204 | SSc_TREAT | GSE55036 |
| GSM1003074 | SSc_ONLY | GSE40839 | GSM1328206 | SSc_TREAT | GSE55036 |
| GSM1003075 | SSc_ONLY | GSE40839 | GSM1328208 | SSc_TREAT | GSE55036 |
| GSM2038267 | SSc_ONLY | GSE76808 | GSM1328209 | SSc_TREAT | GSE55036 |
| GSM2038268 | SSc_ONLY | GSE76808 | GSM1328211 | SSc_TREAT | GSE55036 |
| GSM2038269 | SSc_ONLY | GSE76808 | GSM1328212 | SSc_TREAT | GSE55036 |
| GSM2038270 | SSc_ONLY | GSE76808 | GSM1328214 | SSc_TREAT | GSE55036 |
| GSM2038271 | SSc_ONLY | GSE76808 | GSM1328215 | SSc_TREAT | GSE55036 |
| GSM2038272 | SSc_ONLY | GSE76808 | GSM1328217 | SSc_TREAT | GSE55036 |
| GSM2038273 | SSc_ONLY | GSE76808 | GSM1328218 | SSc_TREAT | GSE55036 |
| GSM2038274 | SSc_ONLY | GSE76808 | GSM1328220 | SSc_TREAT | GSE55036 |
| GSM2038275 | SSc_ONLY | GSE76808 | GSM1328221 | SSc_TREAT | GSE55036 |
| GSM2038276 | SSc_ONLY | GSE76808 | GSM1328222 | SSc_TREAT | GSE55036 |
| GSM2038277 | SSc_ONLY | GSE76808 | GSM1328224 | SSc_TREAT | GSE55036 |
| GSM2038278 | SSc_ONLY | GSE76808 | GSM1328225 | SSc_TREAT | GSE55036 |
| GSM2038279 | SSc_ONLY | GSE76808 | GSM1328226 | SSc_TREAT | GSE55036 |
| GSM2038280 | SSc_ONLY | GSE76808 | GSM1328228 | SSc_TREAT | GSE55036 |
| GSM2149850 | SSc_ONLY | GSE81292 | GSM1328229 | SSc_TREAT | GSE55036 |
| GSM2149851 | SSc_ONLY | GSE81292 | GSM1328230 | SSc_TREAT | GSE55036 |
| GSM2149853 | SSc_ONLY | GSE81292 | GSM1328232 | SSc_TREAT | GSE55036 |
| GSM2149854 | SSc_ONLY | GSE81292 | GSM1328233 | SSc_TREAT | GSE55036 |
| GSM2149855 | SSc_ONLY | GSE81292 | GSM1328235 | SSc_TREAT | GSE55036 |
| GSM2149856 | SSc_ONLY | GSE81292 | GSM1328236 | SSc_TREAT | GSE55036 |
| GSM2149857 | SSc_ONLY | GSE81292 | GSM1328237 | SSc_TREAT | GSE55036 |
| GSM2149858 | SSc_ONLY | GSE81292 | GSM1328239 | SSc_TREAT | GSE55036 |
| GSM2149859 | SSc_ONLY | GSE81292 | GSM1328240 | SSc_TREAT | GSE55036 |
| GSM2149860 | SSc_ONLY | GSE81292 | GSM1328241 | SSc_TREAT | GSE55036 |
| GSM2149861 | SSc_ONLY | GSE81292 | GSM1328243 | SSc_TREAT | GSE55036 |
| GSM2149862 | SSc_ONLY | GSE81292 | GSM1328244 | SSc_TREAT | GSE55036 |
| GSM2149863 | SSc_ONLY | GSE81292 | GSM1328245 | SSc_TREAT | GSE55036 |
| GSM2149864 | SSc_ONLY | GSE81292 | GSM1003058 | WT | GSE40839 |
| GSM1328197 | SSc_ONLY | GSE55036 | GSM1003059 | WT | GSE40839 |
| GSM1328201 | SSc_ONLY | GSE55036 | GSM1003060 | WT | GSE40839 |
| GSM1328205 | SSc_ONLY | GSE55036 | GSM1003061 | WT | GSE40839 |
| GSM1328207 | SSc_ONLY | GSE55036 | GSM1003062 | WT | GSE40839 |
| GSM1328210 | SSc_ONLY | GSE55036 | GSM1003063 | WT | GSE40839 |
| GSM1328213 | SSc_ONLY | GSE55036 | GSM1003065 | WT | GSE40839 |
| GSM1328216 | SSc_ONLY | GSE55036 | GSM1003066 | WT | GSE40839 |
| GSM1328219 | SSc_ONLY | GSE55036 | GSM1003067 | WT | GSE40839 |
| GSM1328223 | SSc_ONLY | GSE55036 | GSM2038281 | WT | GSE76808 |
| GSM1328227 | SSc_ONLY | GSE55036 | GSM2038283 | WT | GSE76808 |
| GSM1328231 | SSc_ONLY | GSE55036 | GSM2038284 | WT | GSE76808 |
| GSM1328234 | SSc_ONLY | GSE55036 | GSM2149865 | WT | GSE81292 |
| GSM1328238 | SSc_ONLY | GSE55036 | GSM2149866 | WT | GSE81292 |
| GSM1328242 | SSc_ONLY | GSE55036 | GSM2149869 | WT | GSE81292 |
| GSM1328246 | SSc_ONLY | GSE55036 |  | | |

**Figure S1. The core compounds connect closely sharing more than 152 targets.**


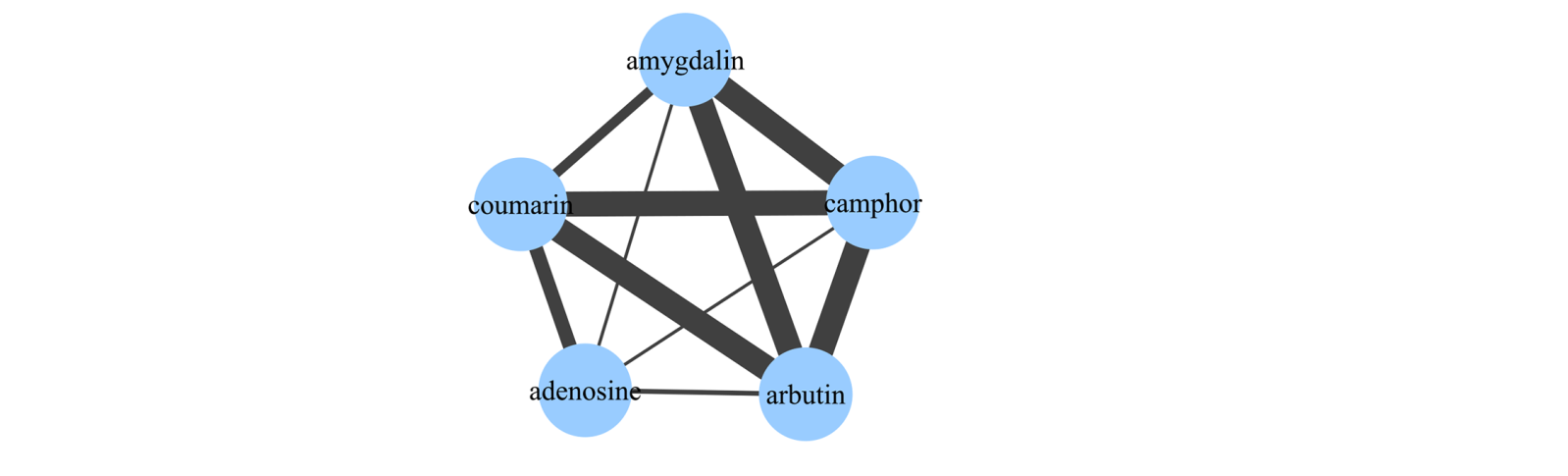


**Figure S2. The PPI network of GFW formula targets, disease genes, drug targets and other targets. T**he network was constructed by ClueGO in Cytoscape and ecomposed into 24 functional modules with a visual plug-in MCODE of Cytoscape. (red: Formula targets; yellow: Disease genes; green: drug targets; orange: Formula targets and disease genes; purple: Formula targets and drug targets; bule: Disease gene and drug targets; pink: Formula targets, drug targets and disease genes; gray: other genes).


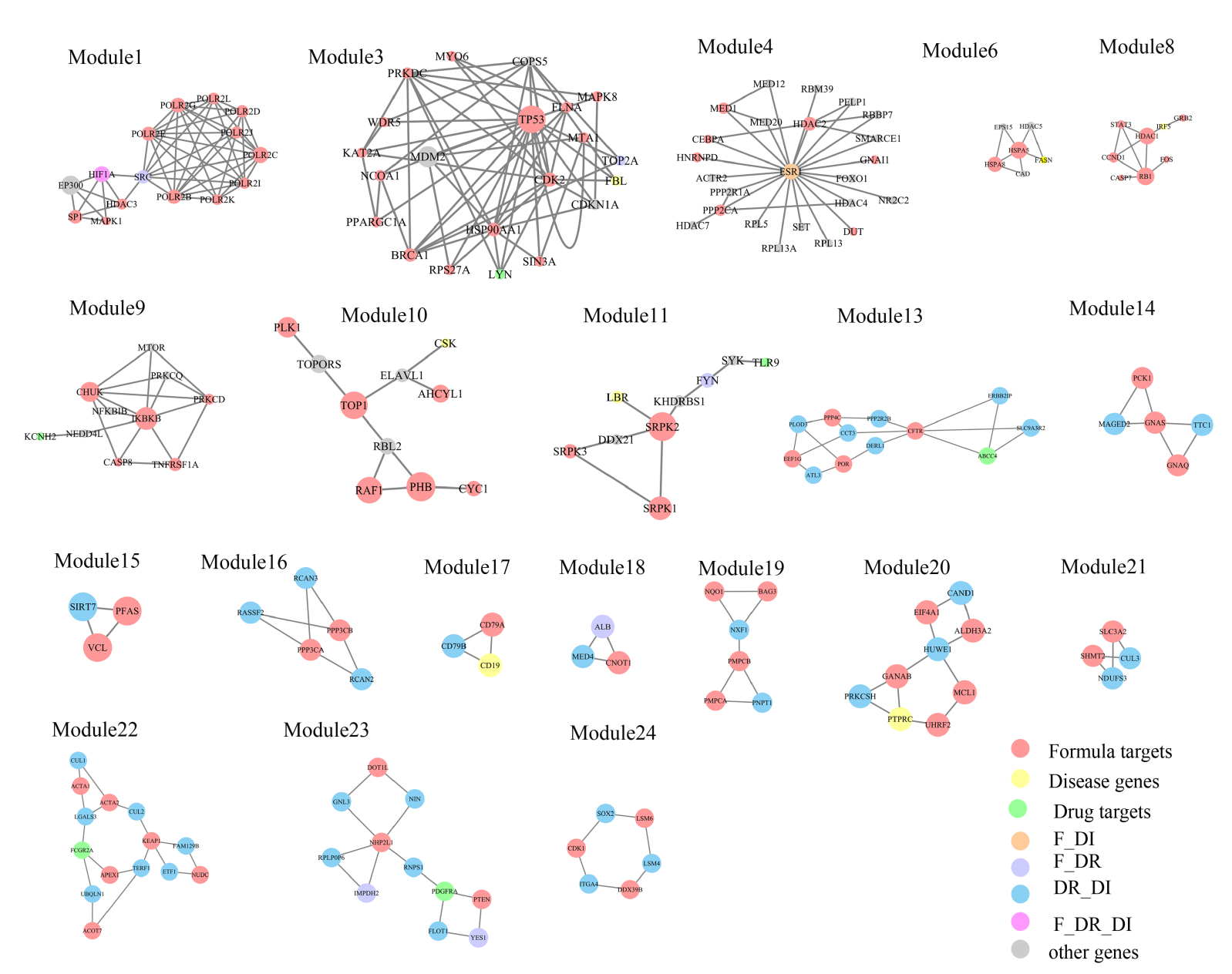

Supplement: Supplementary file 1 — Supplementary Information [file 41598_2018_36314_MOESM1_ESM.docx]
